# Dysregulations of MicroRNA and Gene Expression in Chronic Venous Disease

**DOI:** 10.3390/jcm9051251

**Published:** 2020-04-25

**Authors:** Daniel P. Zalewski, Karol P. Ruszel, Andrzej Stępniewski, Dariusz Gałkowski, Jacek Bogucki, Łukasz Komsta, Przemysław Kołodziej, Paulina Chmiel, Tomasz Zubilewicz, Marcin Feldo, Janusz Kocki, Anna Bogucka-Kocka

**Affiliations:** 1 Chair and Department of Biology and Genetics, Medical University of Lublin, 4a Chodźki St., 20-093 Lublin, Poland; daniel.piotr.zalewski@gmail.com (D.P.Z.); przemyslaw.kolodziej@umlub.pl (P.K.); pachmiel13@gmail.com (P.C.); 2 Chair of Medical Genetics, Department of Clinical Genetics, Medical University of Lublin, 11 Radziwiłłowska St., 20-080 Lublin, Poland; karol.ruszel@umlub.pl (K.P.R.); jacek.bogucki@umlub.pl (J.B.); janusz.kocki@umlub.pl (J.K.); 3 Ecotech Complex Analytical and Programme Centre for Advanced Environmentally Friendly Technologies, University of Marie Curie-Skłodowska, 39 Głęboka St., 20-612 Lublin, Poland; andrzej.stepniewski@umcs.pl; 4 Department of Pathology and Laboratory Medicine, Rutgers-Robert Wood Johnson Medical School, One Robert Wood Johnson Place, New Brunswick, NJ 08903-0019, USA; galkowd@fastmail.fm; 5 Chair and Department of Medicinal Chemistry, Medical University of Lublin, 4 Jaczewskiego St., 20-090 Lublin, Poland; lukasz.komsta@umlub.pl; 6 Chair and Department of Vascular Surgery and Angiology, Medical University of Lublin, 11 Staszica St., 20-081 Lublin, Poland; tomasz.zubilewicz@umlub.pl (T.Z.); martinf@interia.pl (M.F.)

**Keywords:** chronic venous disease, CVD, varicose veins, miRNA, microRNA, gene, expression, next generation sequencing, biomarker

## Abstract

Chronic venous disease (CVD) is a vascular disease of lower limbs with high prevalence worldwide. Pathologic features include varicose veins, venous valves dysfunction and skin ulceration resulting from dysfunction of cell proliferation, apoptosis and angiogenesis. These processes are partly regulated by microRNA (miRNA)-dependent modulation of gene expression, pointing to miRNA as a potentially important target in diagnosis and therapy of CVD progression. The aim of the study was to analyze alterations of miRNA and gene expression in CVD, as well as to identify miRNA-mediated changes in gene expression and their potential link to CVD development. Using next generation sequencing, miRNA and gene expression profiles in peripheral blood mononuclear cells of subjects with CVD in relation to healthy controls were studied. Thirty-one miRNAs and 62 genes were recognized as potential biomarkers of CVD using DESeq2, Uninformative Variable Elimination by Partial Least Squares (UVE-PLS) and ROC (Receiver Operating Characteristics) methods. Regulatory interactions between potential biomarker miRNAs and genes were projected. Functional analysis of microRNA-regulated genes revealed terms closely related to cardiovascular diseases and risk factors. The study shed new light on miRNA-dependent regulatory mechanisms involved in the pathology of CVD. MicroRNAs and genes proposed as CVD biomarkers may be used to develop new diagnostic and therapeutic methods.

## 1. Introduction

Chronic Venous Disease (CVD) is a common vascular disease of the lower limbs. Estimates of the worldwide prevalence of symptomatic CVD range as high as 60% [1,2]. The common risk factors include age, obesity, smoking, low physical activity, periods of prolonged standing or sitting and positive family history [2]. CVD is defined as a syndrome of chronic morphological and functional abnormalities of the venous system, manifested either by symptoms (including tingling, aching, burning, pain, muscle cramps, swelling, sensations of throbbing or heaviness, itching, restless legs, leg tiredness and fatigue) or clinical signs prompting the need for investigation and medical care [3]. The disease encompasses a wide spectrum of clinical presentations such as telangiectasia, varicose veins, leg edema, skin changes and ulcers, as included in the Clinical, Etiology, Anatomic, Pathophysiology (CEAP) classification [4,5,6]. Chronic Venous Insufficiency (CVI) is a term reserved for advanced CVD, which is applied to functional abnormalities of the venous system producing edema, skin changes, or venous ulcers (C3–C6 in CEAP classification) [3].

CVD is caused by hemodynamic disturbances in veins of lower limbs, presenting as venous occlusion, venous valve incompetency and calf muscle pump dysfunction, which lead to venous hypertension and reflux [5,7,8,9]. In the majority of cases, the great and small saphenous veins are involved [8]. The skin manifestations, such as edema, dermatitis, lipodermatosclerosis and ulceration, are the consequence of chronic volume overload and hypertension in subcutaneous veins, as well as inflammatory processes within skin tissues [10,11]. Imbalance between metalloproteinases and their inhibitors as well as accumulation of leukocytes in the tissues surrounding the venous vessels of the lower limbs under high pressure conditions is considered to be the basis for chronic inflammation and pathological changes in the skin tissue of patients with chronic venous insufficiency [7,12,13].

A significant decrease in life quality, caused by pain, reduced mobility and leg ulcerations, affects patients with CVD [14,15].

The most recommended diagnostic procedures of CVD include physical examination and duplex ultrasound scanning, and the most effective treatment options are compression therapy and invasive interventions, complemented by pharmacotherapy [16,17]. New, more effective diagnostic and treatment strategies are needed and more profound understanding of pathology, particularly the interactions between molecular and cellular mechanisms, is essential for development of optimal treatment approaches.

MicroRNA (miRNA) has been the subject of many studies, greatly expanding the knowledge of their diversity and functions [18]. MiRNAs are approximately 18–25-nucleotides long, single-stranded RNAs involved in modulating gene expression pathways [19]. MiRNAs incorporated in protein complex exhibit gene expression regulating effect by binding to mRNA. The pairing effect of miRNA–mRNA interactions reduces gene expression predominantly by repression of translation, destabilization and cleavage of mRNA [20]. MiRNAs exert their effect as a switch and a fine-tuner of gene expression, providing a pleiotropic effect on protein pool in cells [21]. Alterations in genes involved in miRNA processing were found in various human cancers [22]. MiRNAs are intensively studied as potential means of novel diagnostic and treatment approaches [23,24]

A growing amount of evidence suggests a relevant role of miRNA in vascular cell functions, including cell differentiation, proliferation, migration, and apoptosis [25,26]. MiRNAs are involved in vascular diseases, exhibiting modulatory function of angiogenesis, endothelial cells dysfunction and response for ischemic events [27]. Numerous miRNAs are considered as potential markers of cardiovascular diseases (e.g., coronary artery disease, myocardial infarction, atherosclerosis, venous thromboembolism), exhibiting promising diagnostic, prognostic and therapeutic value [27,28,29,30,31].

Altered expression patterns of miRNAs and genes were demonstrated in vein specimens derived from patients with CVI and compared to healthy subjects [32,33]. Dysregulation of miRNA expression, reported in venous ulcers biopsies, has been associated with inhibition of wound healing [34]. The proposed role of miRNA in CVI susceptibility has also been reported [35]. Therefore, miRNAs could also be involved in the pathogenesis of CVD and may be relevant as potential diagnostic and therapeutic targets.

In our work, integrated miRNA and gene expression analysis was applied to find potential robust biomarkers of CVD and to show the impact of miRNA-regulated genes on pathological processes governing CVD development.

## 2. Experimental Section

### 2.1. Study Participants Characteristics

The study was performed in accordance with the Declaration of Helsinki and approved by the Ethics Committee at Medical University of Lublin (approval No. KE-0254/341/2015). Participants were recruited between February 2016 and May 2017. All subjects gave their informed consent for inclusion before they participated in the study. The CVD group consisted of 34 patients diagnosed and hospitalized only due to CVD, without any other diagnosed vascular diseases or comorbidities, in the Independent Public Clinical Hospital No. 1 in Lublin, Poland. The control group comprised of 19 healthy volunteers without any visible CVD characteristics and lack of comorbidities during examination. Detailed characteristics of included participants are presented in Table 1.

Inclusion and exclusion criteria were evaluated by a vascular surgeon. Included CVD patients were examined using tourniquet test, auscultation and duplex ultrasound scanning. Venous reflux lasting longer than 1 s was classified as pathological. Patients diagnosed with symptoms classified according to CEAP as varicose veins (C2) of superficial veins (As) with primary etiology (Ep) and reflux pathophysiology (Pr) were included. The exclusion criteria were previous vascular surgery of lower limbs, insufficiency of deep veins, acute and chronic inflammation of veins, lower extremities arterial disease, coronary artery disease, cerebrovascular disease, aneurismal disease, myocardial infarction, hypertension, stroke, diabetes mellitus type 2, and pregnancy.

Body Mass Index (BMI), pain symptoms, ankle-brachial index, smoking habits and applied medical treatment were also evaluated (Table 1).

The control group consisted of 19 healthy, nonsmoking volunteers (Table 1). Only subjects without blood flow disturbances and with normal morphology of veins in lower limbs, confirmed by physical examination and duplex ultrasound scanning, were included to the study as controls. Any symptoms, comorbidities and treatment of vascular diseases were indicated in the analysis of medical history of control subjects.

### 2.2. Study Material Preparation

Isolation of Peripheral Blood Mononuclear Cells (PBMCs) was performed from whole blood samples by density gradient centrifugation using Gradisol L reagent (Aqua-Med, Łódź, Poland). Small RNA fractions were isolated from PBMCs samples of 34 CVD patients and 19 controls using MirVana microRNA Isolation Kit (Ambion, Austin, TX, USA), according to the manufacturer’s protocol. Total RNA was isolated from PBMCs of seven randomly selected CVD patients and seven randomly selected control subjects, using TRI Reagent Solution (Applied Biosystems, Foster, CA, USA) according to the manufacturer’s protocol. For a more detailed description of study material isolation and assessment refer to [30].

### 2.3. Libraries Preparation and Sequencing

Small RNA libraries were prepared from 53 small RNA samples isolated from PBMCs of 34 CVD patients and 19 healthy controls. Technical limitations did not allow to perform transcriptome sequencing for all subjects included in the study, therefore transcriptome libraries were constructed from 14 total RNA samples isolated from randomly selected, representative subsets of PBMCs samples (seven from CVD patients and seven from healthy controls).

Small RNA and transcriptome libraries were prepared using Ion Total RNA-Seq Kit v2, Magnetic Bead Cleanup Module kit and barcoded with Ion Xpress RNA-Seq Barcode 01-16 Kit (all Life Technologies, Carlsbad, CA, USA), according to the manufacturer’s protocol “Ion Total RNA-Seq Kit v2” revision B.0. Libraries were sequenced on Ion 540 Chips (Life Technologies) using Ion S5 XL System (ThermoFisher Scientific, Waltham, MA, USA). Small RNA and transcriptome raw sequencing data were aligned to 2792 human miRNAs from miRBase v21 (http://www.mirbase.org) and to 55,765 genes and splicing variants of hg19 human genome, respectively.

Detailed description of libraries preparation and sequencing procedures were included in our previous study [30].

### 2.4. Statistical Analysis

The differences between CVD and control groups were evaluated in terms of age and BMI using a two-sided Mann–Whitney *U* test (wilcox.test function in R), and in terms of sex and smoking using Fisher exact test (fisher.test function in R).

Statistical analysis of miRNA and gene expression datasets was performed on biological replicates using R environment (version 3.5.2, https://www.r-project.org) with proper packages. Differential expression analysis was carried out using DESeq2 package v1.18.1 (https://bioconductor.org/packages/release/bioc/html/DESeq2.html) [36]. MiRNAs and genes with *p* value below 0.05 after Benjamini–Hochberg false discovery rate correction were considered as statistically significant. Differential potential of miRNAs and genes was further confirmed by Uninformative Variable Elimination by Partial Least Squares (UVE-PLS) method [37] using the plsVarSel package v0.9.3 (https://cran.r-project.org/web/packages/plsVarSel/index.html) [38].

Venn diagrams, Heatmaps with Euclidean clustering and 3D Principal Component Analysis (PCA) plots were created using VennDiagram 1.6.20 (https://cran.r-project.org/web/packages/VennDiagram/index.html) [39], pheatmap 1.0.10 (https://cran.r-project.org/web/packages/pheatmap/index.html) and scatterplot3d 0.3-41 (https://cran.r-project.org/web/packages/scatterplot3d/index.html) [40] packages, respectively. Correlation analysis was performed using Spearman rank correlation test implemented in the Hmisc package 4.4-0 (https://cran.r-project.org/web/packages/Hmisc/index.html).

A Receiver Operating Characteristics (ROC) analysis implemented in pROC package 1.12.1 [41] (https://cran.r-project.org/web/packages/pROC/index.html) was used to evaluate the predictive value of selected miRNAs and gene transcripts for CVD classification.

Deconvolution of gene expression was performed by “quanTIseq” [42] and “MCPcounter” [43] methods implemented to immunedeconv 2.0.0 package (https://rdrr.io/github/grst/immunedeconv/) [44].

MultiMiR package 1.2.0 (https://bioconductor.org/packages/release/bioc/html/multiMiR.html) [45] was used to identify validated and predicted interactions between selected miRNAs and genes. Visualization of the regulatory network with interactions was performed using Cytoscape v3.5.1 software (https://cytoscape.org/) [46].

Functional analysis of networked genes was performed using Database for Annotation, Visualization and Integrated Discovery (DAVID) 6.8 tool (https://david.ncifcrf.gov/) [47,48]. Default whole genome of *Homo sapiens* was applied as a background. All the terms of Kyoto Encyclopedia of Genes and Genomes (KEGG), Reactome and Genetic Association Database (GAD) databases associated with analyzed genes were harvested. Enrichment analysis of functional terms was proceeded for Gene Ontology (GO) terms separately for up- and downregulated genes.

All statistical procedures applied to this study were previously described in detail in [30]. Statistical analysis and visualizations were performed according to R code available in published reference manuals of used packages.

**Figure 1:**
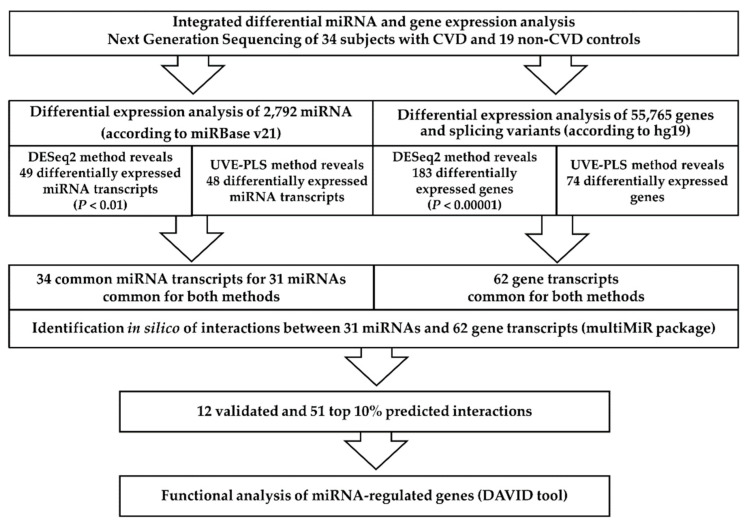
The scheme of the methodology applied in the study. CVD—chronic venous disease.

The summarized methodology applied in our study is presented on Figure 1.

## 3. Results

### 3.1. Study Population Analysis

Characteristics of 34 patients with CVD and 19 non-CVD controls are presented in Table 1. A statistically significant difference between these groups was observed in age (*p* = 8.387 × 10^−3^) and smoking history (*p* = 1.296 × 10^−4^), which probably results from inclusion of healthy CVD-negative non-smoking individuals in control group. No statistically significant differences between CVD and control groups concerning gender and BMI were found (Table 1, Figure S1).

### 3.2. Primary Results

Detailed description of small RNA samples and small RNA libraries as well as the results of primary analysis of small RNA libraries sequencing data are presented in Table S1. Detailed description of transcriptome libraries and results of primary analysis of transcriptome libraries sequencing data are presented in Table S2. Plots depicting sequencing data quality, including boxplot of Cook’s distances, MA plot and histogram of *p* values, regarding small RNA and transcriptome sequencing are presented in Figure S2 and Figure S3, respectively.

### 3.3. Differential Expression Analysis of miRNA

The comparison of miRNA expression levels between 34 CVD patients and 19 non-CVD controls was performed using DESeq2 and UVE-PLS methods and significantly dysregulated miRNAs selected by both methods were chosen.

DESeq2 analysis revealed 1034 differentially expressed miRNA transcripts in CVD subjects compared to controls. Ninety-six miRNA transcripts were differentially expressed with statistical significance *p* < 0.05 (Table S3). DESeq2 method is characterized by relatively high sensitivity, therefore a set of 49 differentially expressed miRNA transcripts (for 41 miRNAs) of higher significance (*p* < 0.01) was selected to limit the number of potentially false positive results.

In order to optimally filter uninformative miRNAs, the UVE-PLS method was applied to miRNA expression data of 1034 differentially expressed miRNA transcripts in CVD subjects. UVE-PLS analysis returned 48 informative miRNA transcripts (Table S4). Figure S4 shows the arrangement of prediction error and PLS components as well as cross-validated predictions versus measured values.

**Figure 2:**
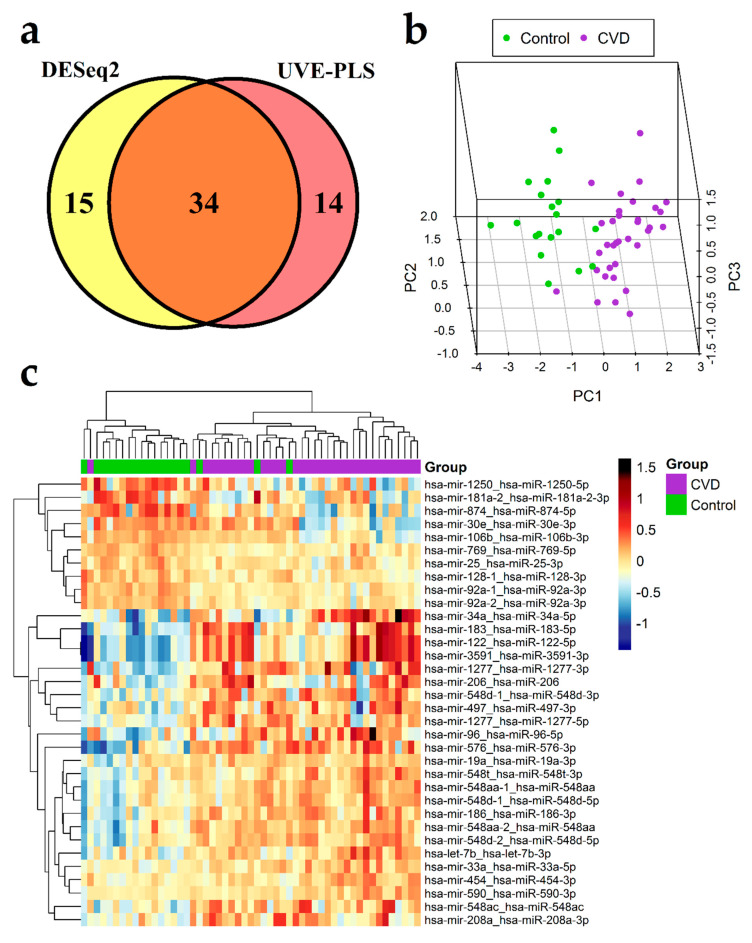
Results of differential expression analysis of miRNA in group of 34 patients with chronic venous disease (CVD) vs. 19 healthy controls (Control). (**a**) Venn diagram presenting comparison of two sets of miRNA transcripts: the set of 49 miRNA transcripts resulted from DESeq2 analysis with *p* < 0.01 and the set of 48 informative miRNA transcripts resulted from Uninformative Variable Elimination by Partial Least Squares (UVE-PLS) analysis. Thirty-four miRNA transcripts were common for both sets. 3D Principal Component Analysis (PCA) plot (**b**) and heatmap with Euclidean clustering (**c**) show differential expression of common 34 miRNA transcripts in CVD and control groups.

The set of 49 differentially expressed miRNA transcripts identified by DESeq2 method (with *p* < 0.01) and the set of 48 differentially expressed miRNA transcripts identified by UVE-PLS method were compared on the Venn diagram, revealing 34 miRNA transcripts common for both sets (Figure 2a). These 34 miRNA transcripts result in 31 miRNAs (22 upregulated and 9 downregulated), which constitute a proposed panel of potential miRNA biomarkers of CVD (Table 2). Differential expression of common 34 miRNA transcripts in CVD and control group is visualized on 3D PCA plot and in heatmap with Euclidean clustering (Figure 2b,c, respectively).

ROC analysis revealed that areas under ROC curves for 34 selected miRNA transcripts were covered in the range 0.930–0.757, indicating a high ability to distinguish patients with CVD from healthy subjects (Table 2, Table S5 and Figure S5).

Correlation analysis between age and expression data of 34 selected miRNA transcripts was performed in CVD group in order to evaluate effect of age on these miRNAs (Table S6). Only hsa-miR-548ac was statistically significantly correlated with age (*p* = 0.0395) exhibiting weak and negative correlation (R = −0.35). The lack of statistically significant correlation of remaining 33 miRNA transcripts suggests their independency from age; however, further studies with larger populations should be performed to confirm this result.

### 3.4. Differential Expression Analysis of Genes

From CVD patient and non-CVD control groups, seven patients and seven controls were randomly selected for gene expression analysis. Similarly to miRNA, DESeq2 and UVE-PLS methods were applied to perform differential gene expression analysis. Significantly dysregulated genes revealed by both methods were selected.

DESeq2 analysis disclosed 23,204 differentially expressed genes in CVD subjects, comparing to controls. In total, 2719 genes presented statistical significance (*p* < 0.05). The risk of false positive results was decreased by selection of 183 differentially expressed genes with *p* < 0.00001 (Table S7).

Application of UVE-PLS analysis to gene expression data of 23,204 differentially expressed genes in CVD subjects compared to controls disclosed 74 informative genes (Table S8). Plot presenting the arrangement of prediction error and PLS components as well as plot of cross-validated predictions versus measured values were shown on Figure S6.

**Figure 3:**
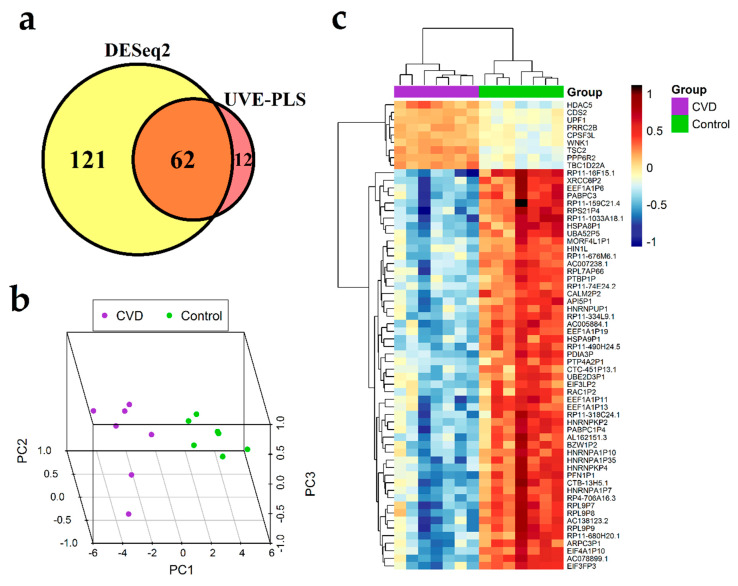
Results of differential expression analysis of genes in group of seven patients with chronic venous disease (CVD) vs. seven healthy controls group (Control). (**a**) Venn diagram presenting comparison of two gene sets: the set of 183 genes received from DESeq2 analysis with *p* < 0.00001 and the set of 74 informative genes indicated by Uninformative Variable Elimination by Partial Least Squares (UVE-PLS) analysis. Sixty-two genes were common for both sets of genes. 3D Principal Component Analysis (PCA) plot (**b**) and heatmap with Euclidean clustering (**c**) show differential expression of common 62 genes in CVD and Control groups.

The set of 183 differentially expressed genes identified by DESeq2 method with *p* < 0.00001 and the set of 74 informative genes identified by UVE-PLS method were compared on a Venn diagram, revealing 62 genes common for both sets (Figure 3a). These 62 common genes constitute a proposed panel of potential biomarkers of CVD (Table 3). Clustering patterns of 62 genes in CVD subjects and controls was visualized on 3D PCA plot and in heatmap with Euclidean clustering (Figure 3b,c, respectively).

The ROC analysis showed that areas under ROC curves obtained for 62 selected genes were equal to 0.98 for *RAC1P2* and *RP11-318C24.1*, and equal to one for the remaining 60 genes, indicating good precision of CVD classification (Table 3, Table S9, Figure S7).

Correlation analysis between age and expression data of 62 selected genes was performed in CVD group in order to evaluate effect of age on these genes (Table S10). *HSPA8P1* (R = −0.82, *p* = 0.024), *PTBP1P* (R = −0.80, *p* = 0.031), *TSC2* (R = −0.79, *p* = 0.033) and *UBA52P5* (R = −0.77, *p* = 0.043) were statistically significantly and negatively correlated with age. Among these four genes, *HSPA8P1*, *PTBP1P* and *UBA52P5* were disclosed as downregulated in CVD group, pointing to them as possible age-associated risk factors of CVD. A lack of statistically significant correlation of remaining 58 genes suggests their independency from age; however, further studies with larger populations should be performed to confirm this result.

To estimate the influence of cell subpopulations diversity in the PBMCs samples on results of gene expression analysis, the deconvolution procedure was carried out using “quanTIseq” and “MCPcounter” methods implemented in immunedeconv package. Both methods enable to perform comparisons between samples and “quanTIseq” allows also to make comparisons between cell types. Application of both methods to the gene expression data showed estimated proportions of 11 cell subpopulations in studied samples (Figures S8 and S9). Although differences in proportions of particular cell subpopulations could be observed between samples, our data suggests that there is no significant impact of cell subpopulations composition in PBMCs samples on the study results.

### 3.5. In Silico Identification of miRNA:Gene Interactions

**Figure 4:**
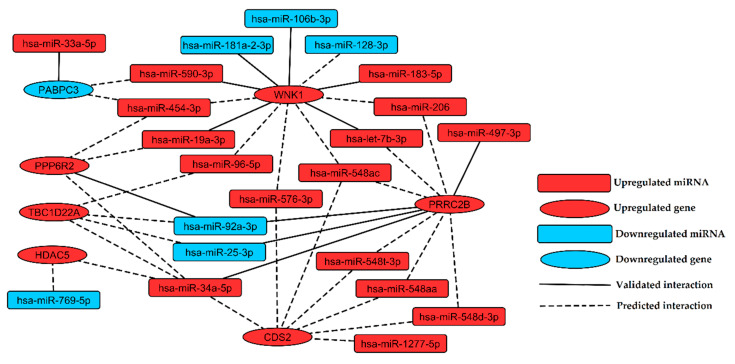
Regulatory network of interactions found in silico between miRNAs and genes indicated as the most promising biomarkers of chronic venous disease. Upregulated and downregulated nodes (miRNAs or genes) were labeled with red and blue color, respectively. Validated and predictive interactions were labeled with solid and dashed edges, respectively.

Identification of miRNA:gene interactions between 31 selected miRNAs and 62 selected genes was performed in silico by multiMiR package. Twelve validated (Table S11) and 51 top 10%-predicted miRNA:gene pairs (Table S12) were returned from analysis. Interactions between miRNAs and their targets were visualized as a regulatory network containing 22 miRNAs and seven genes (Figure 4).

### 3.6. Functional Analysis of miRNA Targets

Functional analysis was performed using DAVID 6.8 tool for seven networked target genes (*CDS2*, *HDAC5*, *PPP6R2*, *PRRC2B*, *TBC1D22A*, *WNK1*, and *PABPC3*). Analyzed genes were associated with cardiovascular diseases and risk factors (*TBC1D22A*, *WNK1*), bone mineral density (*HDAC5*), body weight (*PABPC3*), glycerophospholipid metabolism (*CDS2*), Notch signaling (*HDAC5*), RNA transport and degradation (*PABPC3*). Three genes: *CDS2*, *TBC1D22A*, and *WNK1* were connected to tobacco use disorder, which may be caused by the prevalence of smoking in 14.7% of CVD population (control group consists of non-smoking individuals, Table 1). GO enrichment analysis assigned upregulated genes (*CDS2*, *HDAC5*, *PPP6R2*, *PRRC2B*, *TBC1D22A*, and *WNK1*) to developmental processes and downregulated gene *PABPC3* to RNA metabolic processes (Table 4).

## 4. Discussion

High prevalence, multifactorial character and diverse symptomatology of CVD make this disease one of the major health problems worldwide [49]. There is a lack of sensitive and specific biomarkers for early detection of CVD and for monitoring disease progression. Altered expression of miRNA and its effects on regulation of gene expression make it a promising candidate for novel diagnostic and treatment approaches.

In the presented study, we conducted integrated, comparative analysis of miRNA and gene expression in PBMCs of patients with CVD and healthy controls. We applied Next Generation Sequencing and various statistical and bioinformatic tools to analyze expression profiles of CVD patients vs. controls and to search for genetic signatures of CVD (Figure 1). Most promisingly, 31 miRNAs (Table 2) and 62 genes (Table 3), which potentially may serve as biomarkers for CVD, were selected. The discriminative character of proposed biomarkers was reinforced by decreasing the *p* value of statistical significance in DESeq2 analysis (*p* < 0.01 for miRNAs and *p* < 0.00001 for genes, refer to Table 2 and Table 3, respectively) and elimination of uninformative variables using the UVE-PLS method. In ROC analysis, we confirmed very solid diagnostic value of proposed biomarkers (Table 2 and Table 3, Tables S5 and S9, Figures S5 and S7). This multi-step procedure with strict criteria was applied to obtain scientifically valid results and to eliminate RT-qPCR validation.

The presented miRNA and gene signatures of CVD expand the diagnostic perspective; however, the study population is relatively minor, especially in case of transcriptomic analysis and evaluation with larger cohorts is required to confirm diagnostic utility and discriminative ability of proposed CVD biomarkers.

Integration of miRNA and gene expression analysis in this study allowed us to determine the framework of miRNA regulatory network in CVD, introducing experimentally validated and predictive interactions between significantly differentially expressed miRNA and genes found in patients with CVD (Figure 4). The findings of this study expand our understanding of miRNA functions and provide more insights into a complex network of post-transcriptional control in CVD.

To date, the topic of miRNA and gene expression analysis in venous disorders has not been sufficiently studied [33,34]. Cui et al. used microarray and RT-PCR to research upregulation of miR-34a, miR-202 and downregulation of miR-155 in vein biopsies from patients with CVI [33]. Our study confirmed that upregulated expression of miR-34a is indicative for chronic venous disorder.

Significantly higher expression of miR-16, miR-20a, miR-21, miR-106a, miR-203 and miR-130a was reported in skin biopsies from venous ulcers, comparing to normal skin specimens. Overexpression of these miRNAs was associated with inhibition of wound healing through targeting mRNA for EGR3, Vcl and LepR. Administration of miR-21 mimics in rat acute wound healing models leads to increase in infiltration of immune cells and reduction of epithelialization [34]. In our study, we demonstrated statistically significant upregulation of miR-21 (Table S3) in CVD subjects with varicose veins, which can be classified as an early stage of the disease. Therefore, enhanced signaling of miR-21 appears to be a factor involved in CVD progression both in early and advanced stages of disease development.

In other studies, miR-34a and miR-21 were significantly upregulated in plasma and in atherosclerotic plaques of patients with CAD (Coronary Artery Disease) [50,51], suggesting that overexpression of miR-34a and miR-21 also found in our study to is common for both arterial and venous pathology. Diagnostic and prognostic potential of increased level of miR-21 was also reported as oncomiR in many types of cancer, including Hodgkin lymphoma [52], head and neck squamous cell carcinoma [53] and lung cancer [54,55], while miR-34a acts as tumor suppressor and undergo epigenetic silencing in carcinogenesis [56].

Some studies indicated altered expression of genes in veins obtained from patients with CVD and venous ulcers [57,58,59,60] but none of the genes indicated in abovementioned research were found in our study. These differences could be related to the biological material type, inclusion criteria and methodological approach.

Presented miRNA regulatory network shows that upregulation of *WNK1* is associated with downregulation of miR-181a-2-3p and miR-106b-3p (Figure 4). *WNK1* is important for proper proliferation, chemotaxis and invasion of endothelial cells [61]. Deficiency of the *WNK1* gene in mice induces embryonic lethality due to angiogenic and cardiovascular defects [61]. MiRNAs belonging to miR-181 family play a key role in vascular inflammation through regulation of NF-κB signaling, activation of endothelial cells and homeostasis of immune cells [62]. Upregulation of miR-181a in THP-1 cells exposed to oxidated LDL, lipopolysaccharides and phorbol myristate leads to reduction of foam cells formation and inflammatory cytokines levels, lower production of reactive oxygen species and inhibition of apoptosis [63,64]. A higher level of miR-181a in plasma was proposed as a biomarker of myocardial infarction [65]. Overexpression of miR-106b-3p was previously described in the late stage of endothelial replicative senescence [66]. Downregulation of miR-106b-3p was observed in human retinal pigment epithelium cells (ARPE-19) exposed to hydrogen peroxide (H_2_O_2_) [67]. Therefore, we hypothesize that upregulation of *WNK1* observed in our study, associated with downregulation of miR-181a-2-3p and miR-106b-3p is a possible mechanism promoting inflammation and aging in response to oxidative stress in CVD.

*WNK1* was identified in our study as a putative target for downregulated miR-128-3p (Figure 4). Zhou et al. showed that downregulation of miR-128-3p promotes endothelial cell proliferation through Ca^2+^ and ERK1/2-Akt signaling [68]. Upregulation of *WNK1* as a consequence of miR-128-3p downregulation may constitute the mechanism promoting endothelial cell proliferation in CVD. Downregulation of miR-128-3p could also be a factor alleviating inflammation, since it was reported to stimulate inflammation via targeting the *TNFAIP3* gene and enhancing NF-κB signaling [69].

MiRNA regulatory network of CVD (Figure 4) shows that miR-33a-5p targets *PABPC3*, which is a gene encoding a putative regulator of MEG3 stability [70]. MEG3 is a suppressor of tumor growth, inhibiting proliferation and promoting apoptosis in cancer cells [71] via activation of p53 [72]. Our study points to downregulation of *PABPC3* with accompanying overexpression of miR-33a-5p as a factor which may affect MEG3 stability and in consequence promote cell proliferation in CVD.

Another upregulated miRNA found in CVD subjects, associated with MEG3 is miR-183-5p. This miRNA was reported to alleviate symptoms of hypoxia (such as a decrease in cell viability and migration) in H9c2 rat cardiomyocytes via targeting genes encoding MEG3 and p27 [73]. Thus, the upregulation of miR-183-5p observed in our study may be considered as another factor enhancing cell proliferation and hypertrophy in CVD. Additionally, in the presented miRNA regulatory network, upregulated miR-183-5p interacts with the upregulated *WNK1* gene. This relationship may also lead to a hyper-proliferative effect, since *WNK1* has been reported to be required for proliferation of endothelial cells and vascular smooth muscle cells [61,74].

In our study, downregulation of miR-769-5p in CVD patients was observed. Lower expression of this miRNA was reported in cancer cells and was associated with enhanced proliferation and reduced apoptosis [75,76]. Downregulation of miR-769-5p was also found in arterial samples of abdominal and popliteal arterial aneurysm [77]. In the miRNA regulatory network presented here, *HDAC5* is proposed as a putative target of miR-769-5p. Xu et al. reported that *HDAC5* mediates angiotensin II-induced MEF2 activation and vascular smooth muscle cells hypertrophy [78]. These findings suggest that *HDAC5* upregulation, most likely resulted from downregulation of miR-769-5p, may be responsible for pathological vascular hypertrophy in CVD.

We indicated upregulation of miR-206 in CVD patients as an important target, which was already revealed to impair viability and migration of endothelial progenitor cells and to promote apoptosis through targeting *VEGF* [79]. MiR-206 mediate silencing of *VEGF* leading to inhibition of angiogenesis during *Danio rerio* development [80]. Upregulation of miR-206 in our study may thus point to inhibition of angiogenesis; however, two genes encoding downstream effectors of *VEGF* signaling, *WNK1* [81] and *CDS2* [82], were upregulated in CVD subjects. Upregulation of *WNK1* and *CDS2* may be a consequence of increase in *VEGF* expression mediated by chromatin remodeling caused by upregulation of *HDAC5* [83,84], which was also observed in the current study. These findings may suggest an increase in angiogenesis in CVD independently from the *VEGFR* signaling. On the other hand, a different study indicated *HDAC5* as a negative regulator of angiogenesis acting through genes for pro-angiogenic factors FGF2 and Slit2 [85], showing that reciprocal interaction network in CVD is more complex and requires further studies to answer all questions.

Downregulation of miR-30e-3p was previously observed in coronary microembolization and was associated with myocardial injury mediated by impairment of autophagy in cardiomyocytes [86]. Therefore, the downregulation of miR-30e-3p found in this study may be involved in vascular dysfunction related to the thrombosis in CVD.

Kim and collaborators reported at least 5-fold higher level of miR-33a in plasma from individuals with high risk of atherosclerosis, compared to non-high-risk subjects [87]. A significantly higher level of this miRNA was also described in PBMCs isolates from patients with CAD and was positively correlated with higher lipid levels and risk of atherosclerosis [88,89]. Therefore, the upregulation of miR-33a-5p found in our study may indicate higher risk of atherosclerosis in subjects with CVD, compared to non-CVD subjects.

Upregulation of circulating miR-92a-3p was observed in the plasma of both mild cognitive impairment and Alzheimer disease subjects [90] and was proposed as a biomarker of schizophrenia [91]. In our study, downregulated miR-92a-3p putatively targets upregulated *PRRC2B* (Figure 4), which is a gene encoding protein involved in brain development [92]. It suggests that some elements of neurodevelopmental process may also participate in CVD pathology, showing connections between vascular and neurodegenerative disorders.

The preliminary functional analysis of proposed transcriptomic biomarkers provides useful information on the pathogenesis of CVD. Functional analysis showed the association of three genes (*CDS2*, *TBC1D22A* and *WNK1*) with tobacco use disorder. The discriminative character of these genes in our study may be, to a certain extent, a result of smoking prevalence in the CVD group; however, smoking is also an established risk factor of CVD [2] and altered expression of *CDS2*, *TBC1D22A* and *WNK1* could be involved in CVD development through smoking-related mechanisms. Many smoking-induced miRNAs were previously identified and associated with various pathological conditions, including carcinogenesis [93]. Presence of other smoking-related diseases was limited by inclusion patients diagnosed exclusively for CVD, with no other, especially cardiovascular, conditions detected during examination.

PBMCs constitute an important element of inflammation process in vascular diseases [11]; thus, transcriptional profiling of this cell pool should provide reliable information pertinent for vascular pathology. Another advantage of PBMCs is their accessibility through minimally invasive procedures, facilitating utilization in basic and clinical research. Despite of all advantages, co-prevalence of other diseases like chronic obstructive pulmonary disease, hypertension, diabetes mellitus and accompanying complications may bias the evaluation of expression profiles on a systemic scale. To cope with this bias, only subjects without co-existing conditions mentioned in the experimental section were included to the study. Such strict evaluation helped us to find systemic regulatory changes in miRNA and gene expression, which potentially are reflective of local changes in CVD. However, application of these exclusion criteria entailed construction of CVD and control groups with statistically significant differences in age and smoking history, which can introduce some biological bias to our results; therefore, further investigations with more balanced population groups should be performed.

We are aware that our research has several limitations. Studied PBMCs samples may differ in proportions of cell subpopulations (lymphocytes, monocytes), which may introduce a bias in miRNA and gene expression patterns. To assess the impact of this factor, we performed a deconvolution procedure using two methods: “quanTIseq” and “MCPcounter” implemented in an immunedeconv package, enabling comparison between cell types and between samples. The results of deconvolution do not indicate that differences in proportions of particular cell subpopulations across samples which included the CVD and control group had a significant impact on the study outcome (Figures S8 and S9).

Due to technical constraints (server capacity), gene expression analysis was performed on the subset of participants subjected to miRNA expression analysis, which may affect the described transcriptomic effect of miRNA expression alterations in CVD patients. Despite this imbalance, we confirmed some previously validated interactions (Table S11) and determined with high probability other connections in signaling network (Table S12). Future studies should also include in vitro and in vivo validation of predictive interactions of presented miRNA regulatory network. Moreover, in the group selected for gene expression analysis, both miRNA and total RNA were isolated from PBMC specimens obtained from exactly the same subjects and represent the same physiological conditions probed at the same time and circumstances.

Despite applying strict criteria to select the most promising signatures of CVD, the biomarker role of particular miRNAs and gene sets should be confirmed in further studies with larger cohorts and with the application of other validation methods, such as RT-qPCR. Further experiments will involve investigations regarding clinical factors (e.g., stage of disease or medications) and elucidation of whether dysregulations of miRNAs and genes indicative for CVD were predictive of or responsive to the disease development.

The results obtained in our current study confirm the significance of miRNA-dependent epigenetic regulation in the pathogenesis of CVD. Although we proposed a novel biomarker panel of CVD, there is still a need for further research on the role of miRNA regulation in the CVD due to small sample size as well as a clear exploratory and hypothesis-generating character of the presented experiments.

The presented discoveries may be used in further fundamental research and may prove to be useful for clinicians and practitioners, providing new paths in diagnosis, differentiation and treatment procedures for CVD patients in future.

## 5. Conclusions

Owing to broad and detailed Next Generation Sequencing analysis one is able to draw some general conclusions about CVD. Analysis of microRNAs and genes dysregulated in CVD unveiled numerous terms related to general physiological processes and traits like inflammation, metabolism, aging as well as more specific ones like lipidomics, cardiovascular diseases and chemodependencies. Future research on much numerous groups of patients and controls would broaden our knowledge about cardiovascular diseases, enabling personalized approach to individual patients.

## Figures and Tables

**Table 1:** Characteristics of 34 patients with chronic venous disease (CVD) and 19 non-CVD controls included in the study.

Characteristic	CVD Population (*n* = 34)	Control Population (*n* = 19)	*P*
Age	44.12 ± 10.07 ^1^	36.58 ± 9.97 ^1^	8.387 × 10^−3^
	27–78 ^2^	24–55 ^2^	
Body Mass Index	23.85 ± 2.35 ^1^	23.12 ± 3.93 ^1^	0.117
	20.13–28.76 ^2^	19.33–32.6 ^2^	
Smoking: Current	5 (14.7%)	0 (0%)	1.296 × 10^−4^
Smoking: Former	13 (38%)	0 (0%)	
Smoking: Never	16 (47%)	19 (100%)	
Sex: Male	17 (50%)	9 (47%)	1
Sex: Female	17 (50%)	10 (53%)	
**Signs and symptoms**			
Pain	7 (20.6%)	NA	
Ankle-brachial index	0.96 ± 0.048 ^1^	NA	
	0.71–0.99 ^2^		
**Extended anatomical classification**			
Great saphenous vein (above knee)	23 (67.7%)	NA	
Great saphenous vein (below knee)	7 (20.6%)	NA	
Small saphenous vein	3 (8.8%)	NA	
Great and small saphenous vein	1 (2.9%)	NA	
**Medication**			
Micronized diosmin	19 (55.9%)	NA	
Preparation with vitaminum C, hesperidin and *Ruscus aculeatus* extract	10 (29.4%)	NA	
Both medications	5 (14.7%)	NA	

^1^ mean ± SD, ^2^ range. Statistical significance (*P*) of differences between chronic venous disease (CVD) and control group in age and BMI was calculated using two-sided Mann Whitney *U* test, and in sex and smoking was calculated using Fisher exact test. Inapplicable data were addressed to “NA”.

**Table 2:** Set of 34 differentially expressed miRNA transcripts resulted from DESeq2 analysis with *p* < 0.01 with statistical significance confirmed by UVE-PLS analysis in 34 patients with chronic venous disease compared to 19 controls.

No.	miRNA Transcript	miRNA ID ^1^	*P*	Fold Change	PLS Coefficient	ROC-AUC
**Upregulated miRNA Transcripts**						
1.	hsa-mir-122_hsa-miR-122-5p	hsa-miR-122-5p	1.06 × 10^−9^	2.2135	4.71 × 10^−2^	0.930
2.	hsa-mir-3591_hsa-miR-3591-3p	hsa-miR-3591-3p	1.06 × 10^−9^	2.2127	4.71 × 10^−2^	0.930
3.	hsa-mir-183_hsa-miR-183-5p	hsa-miR-183-5p	2.05 × 10^−6^	1.9316	3.83 × 10^−2^	0.855
4.	hsa-mir-1277_hsa-miR-1277-3p	hsa-miR-1277-3p	2.13 × 10^−5^	1.7727	4.04 × 10^−2^	0.850
5.	hsa-mir-548d-1_hsa-miR-548d-3p	hsa-miR-548d-3p	2.13 × 10^−5^	1.6170	2.09 × 10^−2^	0.859
6.	hsa-mir-34a_hsa-miR-34a-5p	hsa-miR-34a-5p	3.81 × 10^−5^	1.9308	3.45 × 10^−2^	0.847
7.	hsa-mir-576_hsa-miR-576-3p	hsa-miR-576-3p	3.04 × 10^−4^	2.0430	3.21 × 10^−2^	0.842
8.	hsa-mir-454_hsa-miR-454-3p	hsa-miR-454-3p	3.04 × 10^−4^	1.2133	1.05 × 10^−2^	0.833
9.	hsa-mir-548d-1_hsa-miR-548d-5p	hsa-miR-548d-5p	3.44 × 10^−4^	1.3487	1.47 × 10^−2^	0.836
10.	hsa-mir-186_hsa-miR-186-3p	hsa-miR-186-3p	3.61 × 10^−4^	1.3568	1.65 × 10^−2^	0.814
11.	hsa-mir-548d-2_hsa-miR-548d-5p	hsa-miR-548d-5p	3.61 × 10^−4^	1.3498	1.47 × 10^−2^	0.811
12.	hsa-mir-548aa-1_hsa-miR-548aa	hsa-miR-548aa	5.13 × 10^−4^	1.3248	1.46 × 10^−2^	0.819
13.	hsa-mir-548aa-2_hsa-miR-548aa	hsa-miR-548aa	1.02 × 10^−3^	1.3381	1.46 × 10^−2^	0.797
14.	hsa-mir-33a_hsa-miR-33a-5p	hsa-miR-33a-5p	1.02 × 10^−3^	1.2067	1.13 × 10^−2^	0.816
15.	hsa-mir-590_hsa-miR-590-3p	hsa-miR-590-3p	1.02 × 10^−3^	1.1660	6.74 × 10^−3^	0.816
16.	hsa-mir-548t_hsa-miR-548t-3p	hsa-miR-548t-3p	1.81 × 10^−3^	1.3233	8.10 × 10^−3^	0.796
17.	hsa-mir-1277_hsa-miR-1277-5p	hsa-miR-1277-5p	1.84 × 10^−3^	1.3291	2.13 × 10^−2^	0.811
18.	hsa-let-7b_hsa-let-7b-3p	hsa-let-7b-3p	2.06 × 10^−3^	1.3223	1.09 × 10^−2^	0.791
19.	hsa-mir-96_hsa-miR-96-5p	hsa-miR-96-5p	3.73 × 10^−3^	2.2914	2.64 × 10^−2^	0.786
20.	hsa-mir-548ac_hsa-miR-548ac	hsa-miR-548ac	5.53 × 10^−3^	1.7613	2.87 × 10^−2^	0.807
21.	hsa-mir-19a_hsa-miR-19a-3p	hsa-miR-19a-3p	5.82 × 10^−3^	1.1944	8.38 × 10^−3^	0.757
22.	hsa-mir-206_hsa-miR-206	hsa-miR-206	8.00 × 10^−3^	2.0356	2.76 × 10^−2^	0.759
23.	hsa-mir-497_hsa-miR-497-3p	hsa-miR-497-3p	9.31 × 10^−3^	1.4368	1.63 × 10^−2^	0.782
24.	hsa-mir-208a_hsa-miR-208a-3p	hsa-miR-208a-3p	9.81 × 10^−3^	3.2080	2.77 × 10^−2^	0.789
**Downregulated miRNA transcripts**						
25.	hsa-mir-92a-1_hsa-miR-92a-3p	hsa-miR-92a-3p	7.89 × 10^−5^	0.8323	−1.40 × 10^−2^	0.856
26.	hsa-mir-874_hsa-miR-874-5p	hsa-miR-874-5p	1.29 × 10^−4^	0.5428	−3.43 × 10^−2^	0.916
27.	hsa-mir-106b_hsa-miR-106b-3p	hsa-miR-106b-3p	2.47 × 10^−4^	0.7964	−1.15 × 10^−2^	0.902
28.	hsa-mir-92a-2_hsa-miR-92a-3p	hsa-miR-92a-3p	3.04 × 10^−4^	0.8414	−1.43 × 10^−2^	0.842
29.	hsa-mir-181a-2_hsa-miR-181a-2-3p	hsa-miR-181a-2-3p	1.02 × 10^−3^	0.6772	−3.24 × 10^−2^	0.793
30.	hsa-mir-128-1_hsa-miR-128-3p	hsa-miR-128-3p	2.67 × 10^−3^	0.8504	−7.84 × 10^−3^	0.777
31.	hsa-mir-769_hsa-miR-769-5p	hsa-miR-769-5p	5.53 × 10^−3^	0.8706	−1.15 × 10^−2^	0.794
32.	hsa-mir-30e_hsa-miR-30e-3p	hsa-miR-30e-3p	5.53 × 10^−3^	0.7400	−1.51 × 10^−2^	0.805
33.	hsa-mir-1250_hsa-miR-1250-5p	hsa-miR-1250-5p	8.56 × 10^−3^	0.6186	−3.32 × 10^−2^	0.803
34.	hsa-mir-25_hsa-miR-25-3p	hsa-miR-25-3p	8.94 × 10^−3^	0.8603	−9.00 × 10^−3^	0.766

^1^ According to miRBase 22 (http://www.mirbase.org/). These 34 miRNA transcripts result in 31 miRNAs (miRNA IDs). *P* (FDR with Benjamini–Hochberg correction) and fold change values were obtained from DESeq2 analysis. Partial Least Squares (PLS) coefficients were obtained from Uninformative Variable Elimination by Partial Least Squares (UVE-PLS) analysis. Areas under Receiver Operating Characteristics (ROC) curves (ROC-AUC) were received from ROC analysis. MiRNA transcripts were ordered according to increasing *p* values across groups of upregulated and downregulated miRNA transcripts.

**Table 3:** The set of 62 differentially expressed genes in seven patients with chronic venous disease vs. seven controls, resulted from DESeq2 analysis (*p* < 0.00001) with statistical significance confirmed by Uninformative Variable Elimination by Partial Least Squares (UVE-PLS) analysis.

No.	Gene Symbol	Gene Name	*p* Value	Fold Change	PLS Coefficient	ROC-AUC
**Upregulated Genes**						
1.	*TSC2*	TSC complex subunit 2	4.87 × 10^−17^	1.437	8.197 × 10^−4^	1.000
2.	*TBC1D22A*	TBC1 domain family member 22A	4.36 × 10^−11^	1.431	7.572 × 10^−4^	1.000
3.	*PPP6R2*	protein phosphatase 6 regulatory subunit 2	9.52 × 10^−9^	1.361	6.225 × 10^−4^	1.000
4.	*UPF1*	UPF1, RNA helicase and ATPase	2.82 × 10^−7^	1.247	5.077 × 10^−4^	1.000
5.	*WNK1*	WNK lysine deficient protein kinase 1	4.59 × 10^−7^	1.258	4.134 × 10^−4^	1.000
6.	*CDS2*	CDP-diacylglycerol synthase 2	5.31 × 10^−7^	1.241	4.756 × 10^−4^	1.000
7.	*PRRC2B*	proline rich coiled-coil 2B	1.56 × 10^−6^	1.273	4.693 × 10^−4^	1.000
8.	*HDAC5*	histone deacetylase 5	4.89 × 10^−6^	1.432	5.694 × 10^−4^	1.000
9.	*INTS11* (*CPSF3L*)	integrator complex subunit 11	5.95 × 10^−6^	1.246	4.683 × 10^−4^	1.000
**Downregulated genes**						
10.	*AC078899.1*	Unmatched	1.18 × 10^−13^	0.393	−1.586 × 10^−3^	1.000
11.	*RP11-16F15.1*	Unmatched	1.18 × 10^−13^	0.327	−2.068 × 10^−3^	1.000
12.	*EEF1A1P19*	eukaryotic translation elongation factor 1 alpha 1 pseudogene 19	8.40 × 10^−13^	0.500	−1.305 × 10^−3^	1.000
13.	*PFN1P1*	profilin 1 pseudogene 1	4.04 × 10^−11^	0.367	−1.457 × 10^−3^	1.000
14.	*RP4-706A16.3*	Unmatched	4.36 × 10^−11^	0.455	−1.394 × 10^−3^	1.000
15.	*AC005884.1*	Unmatched	4.36 × 10^−11^	0.401	−1.488 × 10^−3^	1.000
16.	*CALM2P2*	calmodulin 2 pseudogene 2	4.36 × 10^−11^	0.386	−1.498 × 10^−3^	1.000
17.	*HSPA8P1*	heat shock protein family A (Hsp70) member 8 pseudogene 1	4.36 × 10^−11^	0.379	−1.575 × 10^−3^	1.000
18.	*RP11-490H24.5*	Unmatched	4.36 × 10^−11^	0.312	−1.508 × 10^−3^	1.000
19.	*EIF4A1P10*	eukaryotic translation initiation factor 4A1 pseudogene 10	4.66 × 10^−11^	0.461	−1.286 × 10^−3^	1.000
20.	*RP11-1033A18.1*	Unmatched	7.00 × 10^−11^	0.381	−1.495 × 10^−3^	1.000
21.	*EIF3FP3*	eukaryotic translation initiation factor 3 subunit F pseudogene 3	1.35 × 10^−10^	0.443	−1.423 × 10^−3^	1.000
22.	*PDIA3P1* (*PDIA3P*)	protein disulfide isomerase family A member 3 pseudogene 1	2.38 × 10^−10^	0.465	−1.240 × 10^−3^	1.000
23.	*HSPA9P1*	heat shock protein family A (Hsp70) member 9 pseudogene 1	2.76 × 10^−10^	0.420	−1.414 × 10^−3^	1.000
24.	*AC007238.1*	Unmatched	3.62 × 10^−10^	0.422	−1.398 × 10^−3^	1.000
25.	*HNRNPA1P7*	heterogeneous nuclear ribonucleoprotein A1 pseudogene 7	3.72 × 10^−10^	0.462	−1.232 × 10^−3^	1.000
26.	*RP11-159C21.4*	Unmatched	4.81 × 10^−10^	0.390	−1.552 × 10^−3^	1.000
27.	*PABPC3*	poly(A) binding protein cytoplasmic 3	1.70 × 10^−9^	0.414	−1.468 × 10^−3^	1.000
28.	*RP11-74E24.2*	Unmatched	1.94 × 10^−9^	0.537	−1.067 × 10^−3^	1.000
29.	*EEF1A1P6*	eukaryotic translation elongation factor 1 alpha 1 pseudogene 6	1.94 × 10^−9^	0.441	−1.375 × 10^−3^	1.000
30.	*XRCC6P2*	X-ray repair cross complementing 6 pseudogene 2	2.89 × 10^−9^	0.373	−1.535 × 10^−3^	1.000
31.	*HNRNPKP2*	heterogeneous nuclear ribonucleoprotein K pseudogene 2	3.13 × 10^−9^	0.424	−1.163 × 10 ^-3	1.000
32.	*EEF1A1P11*	eukaryotic translation elongation factor 1 alpha 1 pseudogene 11	8.40 × 10^−9^	0.448	−1.369 × 10^−3^	1.000
33.	*UBA52P5*	ubiquitin A-52 residue ribosomal protein fusion product 1 pseudogene 5	8.40 × 10^−9^	0.397	−1.306 × 10^−3^	1.000
34.	*RPL9P7*	ribosomal protein L9 pseudogene 7	9.10 × 10^−9^	0.414	−1.417 × 10^−3^	1.000
35.	*RPS21P4*	ribosomal protein S21 pseudogene 4	1.37 × 10^−8^	0.376	−1.531 × 10^−3^	1.000
36.	*RP11-334L9.1*	Unmatched	1.37 × 10^−8^	0.333	−1.206 × 10^−3^	1.000
37.	*HNRNPKP4*	heterogeneous nuclear ribonucleoprotein K pseudogene 4	1.38 × 10^−8^	0.462	−1.120 × 10^−3^	1.000
38.	*RPL9P9*	ribosomal protein L9 pseudogene 9	1.38 × 10^−8^	0.418	−1.302 × 10^−3^	1.000
39.	*AC138123.2*	Unmatched	1.38 × 10^−8^	0.407	−1.422 × 10^−3^	1.000
40.	*HNRNPA1P10*	heterogeneous nuclear ribonucleoprotein A1 pseudogene 10	1.39 × 10^−8^	0.475	−1.227 × 10^−3^	1.000
41.	*MORF4L1P1*	mortality factor 4 like 1 pseudogene 1	3.98 × 10^−8^	0.535	−1.045 × 10^−3^	1.000
42.	*RP11-676M6.1*	Unmatched	8.19 × 10^−8^	0.498	−1.208 × 10^−3^	1.000
43,	*RPL7AP66*	ribosomal protein L7a pseudogene 66	9.71 × 10^−8^	0.485	−1.089 × 10^−3^	1.000
44.	*RP11-680H20.1*	Unmatched	9.99 × 10^−8^	0.411	−1.155 × 10^−3^	1.000
45.	*CTB-13H5.1*	Unmatched	1.41 × 10^−7^	0.418	−1.175 × 10^−3^	1.000
46.	*HNRNPA1P35*	heterogeneous nuclear ribonucleoprotein A1 pseudogene 35	1.49 × 10^−7^	0.350	−1.223 × 10^−3^	1.000
47.	*PTBP1P*	polypyrimidine tract binding protein 1 pseudogene	1.53 × 10^−7^	0.443	−1.095 × 10^−3^	1.000
48.	*API5P1*	apoptosis inhibitor 5 pseudogene 1	1.57 × 10^−7^	0.347	−1.204 × 10^−3^	1.000
49.	*UBE2D3P1*	ubiquitin conjugating enzyme E2 D3 pseudogene 1	1.69 × 10^−7^	0.485	−8.801 × 10^−4^	1.000
50.	*AL162151.3*	Unmatched	1.94 × 10^−7^	0.431	−1.258 × 10^−3^	1.000
51.	*RPL9P8*	ribosomal protein L9 pseudogene 8	2.34 × 10^−7^	0.446	−1.253 × 10^−3^	1.000
52.	*EEF1A1P13*	eukaryotic translation elongation factor 1 alpha 1 pseudogene 13	2.51 × 10^−7^	0.521	−1.211 × 10^−3^	1.000
53.	*PABPC1P4*	poly(A) binding protein cytoplasmic 1 pseudogene 4	2.60 × 10^−7^	0.465	−1.031 × 10^−3^	1.000
54.	*HNRNPUP1*	heterogeneous nuclear ribonucleoprotein U pseudogene 1	2.73 × 10^−7^	0.441	−1.106 × 10^−3^	1.000
55.	*ARPC3P1*	actin related protein 2/3 complex subunit 3 pseudogene 1	3.72 × 10^−7^	0.331	−1.272 × 10^−3^	1.000
56.	*PTP4A2P1*	protein tyrosine phosphatase type IVA, member 2 pseudogene 1	4.59 × 10^−7^	0.500	−9.014 × 10^−4^	1.000
57.	*CTC-451P13.1*	Unmatched	4.77 × 10^−7^	0.513	−9.263 × 10^−4^	1.000
58.	*BZW1P2*	basic leucine zipper and W2 domains 1 pseudogene 2	7.97 × 10^−7^	0.445	−9.598 × 10^−4^	1.000
59.	*RP11-318C24.1*	Unmatched	1.94 × 10^−6^	0.314	−1.062 × 10^−3^	0.980
60.	*OTUD4P1* (*HIN1L*)	OTUD4 pseudogene 1	2.08 × 10^−6^	0.480	−9.962 × 10^−4^	1.000
61.	*EIF3LP2*	eukaryotic translation initiation factor 3 subunit L pseudogene 2	2.33 × 10^−6^	0.457	−9.995 × 10^−4^	1.000
62.	*RAC1P2*	Rac family small GTPase 1 pseudogene 2	3.29 × 10^−6^	0.489	−8.192 × 10^−4^	0.980

*P* (FDR with Benjamini–Hochberg correction) and fold change values were obtained from DESeq2 analysis. PLS coefficients were obtained from UVE-PLS analysis. Areas under Receiver Operating Characteristics (ROC) curves (ROC-AUC) were received from ROC analysis. Genes were ordered according to increasing *p* values across groups of upregulated and downregulated genes. Genes without names assigned by HUGO Multi-symbol checker were termed as “Unmatched”. Synonyms or previous gene symbols were put into brackets.

**Table 4:** Results of functional analysis of seven genes selected in silico as targets of miRNA identified as signatures of chronic venous disease.

Functional Analysis of Upregulated Genes (*CDS2, HDAC5, PPP6R2, PRRC2B, TBC1D22A, WNK1*)	
*KEGG, Reactome, GAD and GAD Class*	
*CDS2*	KEGG: Glycerophospholipid metabolism, Phosphatidylinositol signaling system, Metabolic pathways
	Reactome: Synthesis of PG (Phosphatidylglycerol)
	GAD: Type 2 Diabetes|edema|rosiglitazone, Tobacco Use Disorder
	GAD Class: pharmacogenomic, chemdependency
*HDAC5*	KEGG: Alcoholism, Viral carcinogenesis,
	Reactome: NOTCH1 Intracellular Domain Regulates Transcription, Constitutive Signaling by NOTCH1 PEST Domain Mutants, Constitutive Signaling by NOTCH1 HD + PEST Domain Mutants
	GAD: antidepressant response, Bone Density, Bone mineral density (hip), Bone mineral density (spine), bronchodilator response, Fractures, Bone, Type 2 Diabetes| edema | rosiglitazone
	GAD Class: immune, metabolic, pharmacogenomic
*PPP6R2*	No information
*PRRC2B*	No information
*TBC1D22A*	GAD: Albumins, Arteries, Attention Deficit Disorder with Hyperactivity, Blood Pressure, Body Mass Index, Body Weight, Breath Tests, Cardiomegaly, Cholesterol, Erythrocyte Count, Fibrinogen, Heart Failure, Heart Rate, Leukocyte Count, longevity, Metabolism, Myocardial Infarction, Parkinson Disease, Resistin, Stroke, Thyrotropin, Tobacco Use Disorder, Waist Circumference, Waist-Hip Ratio
	GAD Class: aging, cardiovascular, chemdependency, hematological, immune, metabolic, neurological, other, psych
*WNK1*	Reactome: Stimuli-sensing channels
	GAD: Apoplexy|Brain Ischemia|Stroke, blood pressure, arterial, Chronic renal failure|Kidney Failure, Chronic, Essential Hypertension, Hereditary Sensory and Autonomic Neuropathies, HIV Infections|[X]Human immunodeficiency virus disease, hypertension, null, Tobacco Use Disorder, Type 2 Diabetes| edema | rosiglitazone
	GAD Class: cardiovascular, chemdependency, infection, neurological, pharmacogenomic, renal, unknown
*Gene Ontology terms associated with EASE score <0.1*	
GO Biological Process	cellular developmental process, positive regulation of molecular function
GO Molecular Function	enzyme binding
Functional analysis of downregulated gene (*PABPC3*)	
*KEGG, Reactome, GAD and GAD Class*	
*PABPC3*	KEGG: RNA transport, mRNA surveillance pathway, RNA degradation
	GAD: Body Mass Index, Body Weight, Body Weight Changes, Glomerular Filtration Rate
	GAD Class: metabolic, renal
*Gene Ontology terms associated with PABPC3*	
GO Biological Process	nucleobase-containing compound metabolic process, cellular aromatic compound metabolic process, nitrogen compound metabolic process, metabolic process, cellular process, RNA metabolic process, mRNA metabolic process, cellular nitrogen compound metabolic process, macromolecule metabolic process, cellular metabolic process, primary metabolic process, cellular macromolecule metabolic process, heterocycle metabolic process, organic substance metabolic process, nucleic acid metabolic process, organic cyclic compound metabolic process
GO Molecular Function	nucleotide binding, nucleic acid binding, RNA binding, single-stranded RNA binding, binding, poly(A) binding, small molecule binding, poly-purine tract binding, organic cyclic compound binding, nucleoside phosphate binding, heterocyclic compound binding
GO Cellular Component	extracellular region, intracellular, cell, cytoplasm, vesicle, membrane-bounded vesicle, organelle, membrane-bounded organelle, extracellular organelle, extracellular region part, intracellular part, cell part, extracellular exosome, extracellular vesicle

Analysis was carried out with DAVID 6.8 website tool and all associated functional terms of Kyoto Encyclopedia of Genes and Genomes (KEGG), Reactome, Genetic Association Database (GAD), Genetic Association Database Class (GAD Class) categories are presented. Gene Ontology (GO) terms associated with upregulated genes with EASE score (*p*) < 0.1 are presented. For downregulated gene, all associated GO terms were presented.

## Data Availability

All datasets generated for this study can be found in the FigShare repository https://doi.org/10.6084/m9.figshare.12033738.v1.

## Supplementary Materials

The following are available online at https://www.mdpi.com/2077-0383/9/5/1251/s1, Figure S1: Distribution of age (A), BMI (B), sex (C) and smoking (D) in CVD and control group. Figure S2: Control plots for miRNA sequencing data. Figure S3: Control plots for transcriptome sequencing data. Figure S4: Plots for UVE-PLS analysis of miRNA expression in CVD group in comparison to control group. Figure S5: Receiver Operating Characteristics (ROC) curves for 34 miRNA transcripts selected as signatures of CVD. Figure S6: Plots for UVE-PLS analysis of genes expression in CVD group compared to control group. Figure S7: Receiver Operating Characteristics (ROC) curves for 62 genes selected as signatures of CVD. Figure S8: Results of gene expression deconvolution procedure for seven CVD patients and seven control subjects performed using “quanTIseq” method implemented to immunedeconv 2.0.0 package. Figure S9: Results of deconvolution procedure performed on gene expression datasets of 7 CVD patients (CVD) and 7 control subjects (Control) using “MCPcounter” method implemented to immunedeconv 2.0.0 package. Table S1: Measurements of small RNA samples and small RNA libraries as well as results of small RNA sequencing data analysis received from Ion Torrent small RNA Plugin v5.0.5r3. Table S2: Measurements of transcriptome libraries and results of transcriptome sequencing data analysis received from Ion Torrent RNASeqAnalysis plugin v.5.0.3.0. Table S3: The set of 96 differentially expressed microRNA transcripts resulted from DESeq2 analysis with *p* < 0.05 in 34 patients with CVD compared to 19 controls. Table S4: The set of 48 differentially expressed microRNA transcripts resulted from UVE-PLS analysis in in 34 patients with CVD group compared to 19 controls. Table S5: Results of ROC analysis for 34 miRNA transcripts selected as indicative for CVD. Table S6: Correlation analysis between age and expression of 34 selected miRNA transcripts in CVD group. Table S7: 183 differentially expressed genes resulted from DESeq2 analysis with *p* < 0.00001 in seven patients with CVD, compared to seven controls. Table S8: The set of 74 differentially expressed genes resulted from UVE-PLS analysis in seven patients with CVD compared to seven controls. Table S9: Results of ROC analysis for 62 genes selected as indicative for CVD. Table S10: Correlation analysis between age and expression of 62 selected genes in CVD group. Table S11: Twelve experimentally validated miRNA:gene pairs found among 31 miRNAs and 62 genes indicative for CVD using multiMiR package. Table S12: Top 10%-predicted miRNA:gene pairs found among 31 miRNAs and 62 genes indicative for CVD using multiMiR package.
