# Supplementary material for: Dysregulations of MicroRNA and Gene Expression in Chronic Venous Disease
Source: J Clin Med. 2020 Apr 25;9(5):1251. doi: 10.3390/jcm9051251 (PMC7287878; doi:10.3390/jcm9051251)
Supplement: Supplementary file 1 [file jcm-09-01251-s001.pdf]

SUPPLEMENTARY MATERIAL

Figure S1: Distribution of age (A), BMI (B), sex (C) and smoking (D) in CVD and control group.

Figure S2: Control plots for miRNA sequencing data.

Figure S3: Control plots for transcriptome sequencing data.

Figure S4: Plots for UVE-PLS analysis of miRNA expression in CVD group in comparison to control group.

Figure S5: Receiver Operating Characteristics (ROC) curves for 34 miRNA transcripts selected as signatures of CVD.

Figure S6: Plots for UVE-PLS analysis of genes expression in CVD group compared to control group.

Figure S7: Receiver Operating Characteristics (ROC) curves for 62 genes selected as signatures of CVD.

Figure S8: Results of gene expression deconvolution procedure for seven CVD patients and seven control subjects performed using "quanTIseq" method implemented to immunedeconv 2.0.0 package.

Figure S9: Results of deconvolution procedure performed on gene expression datasets of 7 CVD patients (CVD) and 7 control subjects (Control) using "MCPcounter" method implemented to immunedeconv 2.0.0 package.

Table S1: Measurements of small RNA samples and small RNA libraries as well as results of small RNA sequencing data analysis received from Ion Torrent small RNA Plugin v5.0.5r3.

Table S2: Measurements of transcriptome libraries and results of transcriptome sequencing data analysis received from Ion Torrent RNASeqAnalysis plugin v.5.0.3.0.

Table S3: The set of 96 differentially expressed microRNA transcripts resulted from DESeq2 analysis with $p < 0.05$ in 34 patients with CVD compared to 19 controls.

Table S4: The set of 48 differentially expressed microRNA transcripts resulted from UVE-PLS analysis in in 34 patients with CVD group compared to 19 controls.

Table S5: Results of ROC analysis for 34 miRNA transcripts selected as indicative for CVD. Table S6: Correlation analysis between age and expression of 34 selected miRNA transcripts in CVD group.

Table S7: 183 differentially expressed genes resulted from DESeq2 analysis with $p < 0.00001$ in seven patients with CVD, compared to seven controls.

Table S8: The set of 74 differentially expressed genes resulted from UVE-PLS analysis in seven patients with CVD compared to seven controls.

Table S9: Results of ROC analysis for 62 genes selected as indicative for CVD.

Table S10: Correlation analysis between age and expression of 62 selected genes in CVD group.

Table S11: Twelve experimentally validated miRNA:gene pairs found among 31 miRNAs and 62 genes indicative for CVD using multiMiR package.

Table S12: Top 10%-predicted miRNA:gene pairs found among 31 miRNAs and 62 genes indicative for CVD using multiMiR package.

Supplementary Figures

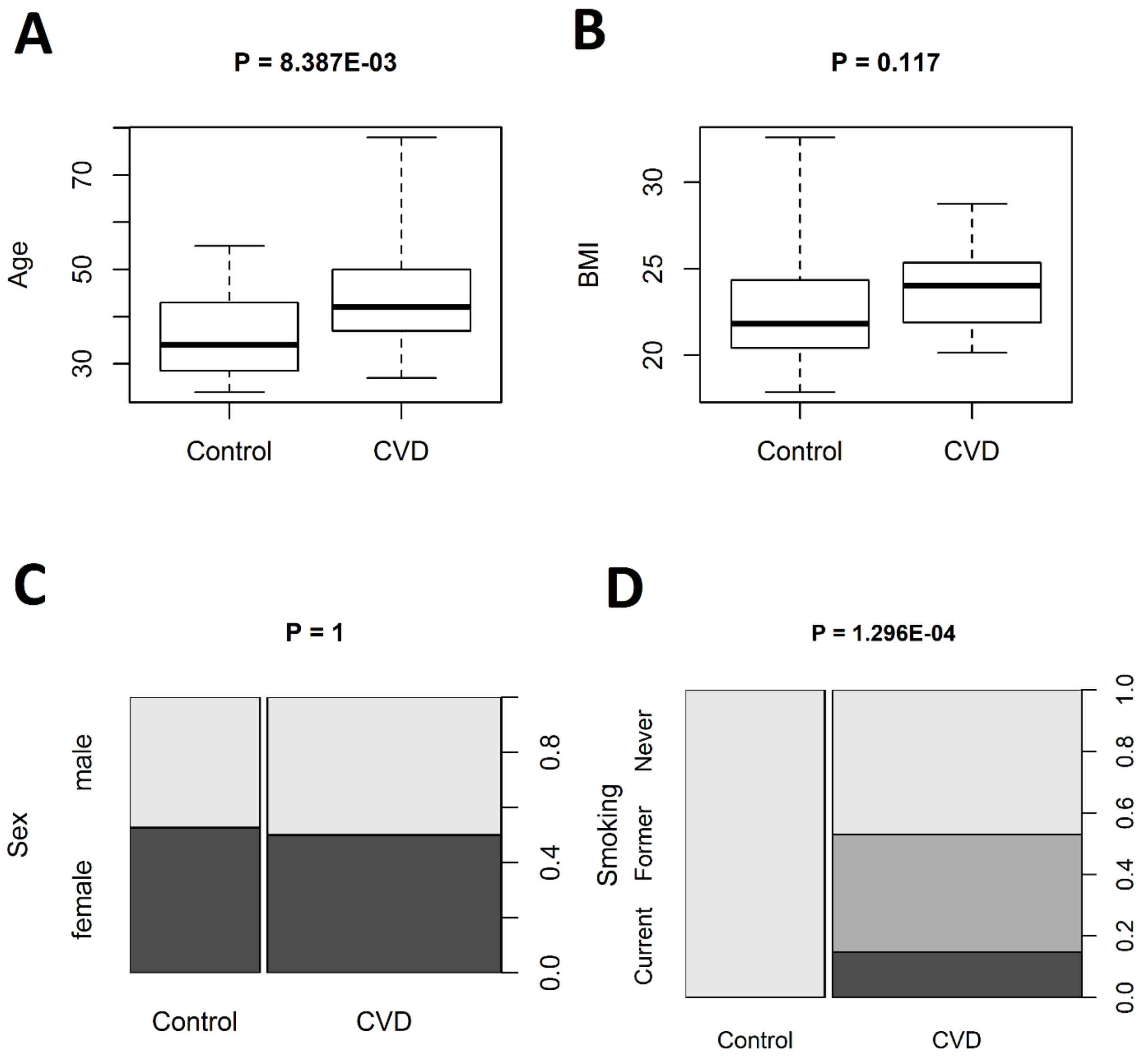

Figure S1. Distribution of age **(A)**, BMI **(B)**, sex **(C)** and smoking **(D)** in CVD and control group. Statistical significance (*P* values) was evaluated using two-sided Mann-Whitney U test (for age and BMI) and two-sided Fisher exact test (for sex and smoking). In the boxplots, whiskers define region between minimum and maximum values, boxes cover values between 25% and 75% quantile and horizontal lines inside boxes mark median value. Spine plot (**C)** presents percentage distribution of sex (black – female, gray – male) and spine plot (**D)** presents percentage distribution of smoking (black – current smokers, medium grey – former smokers, light grey – non-smokers).

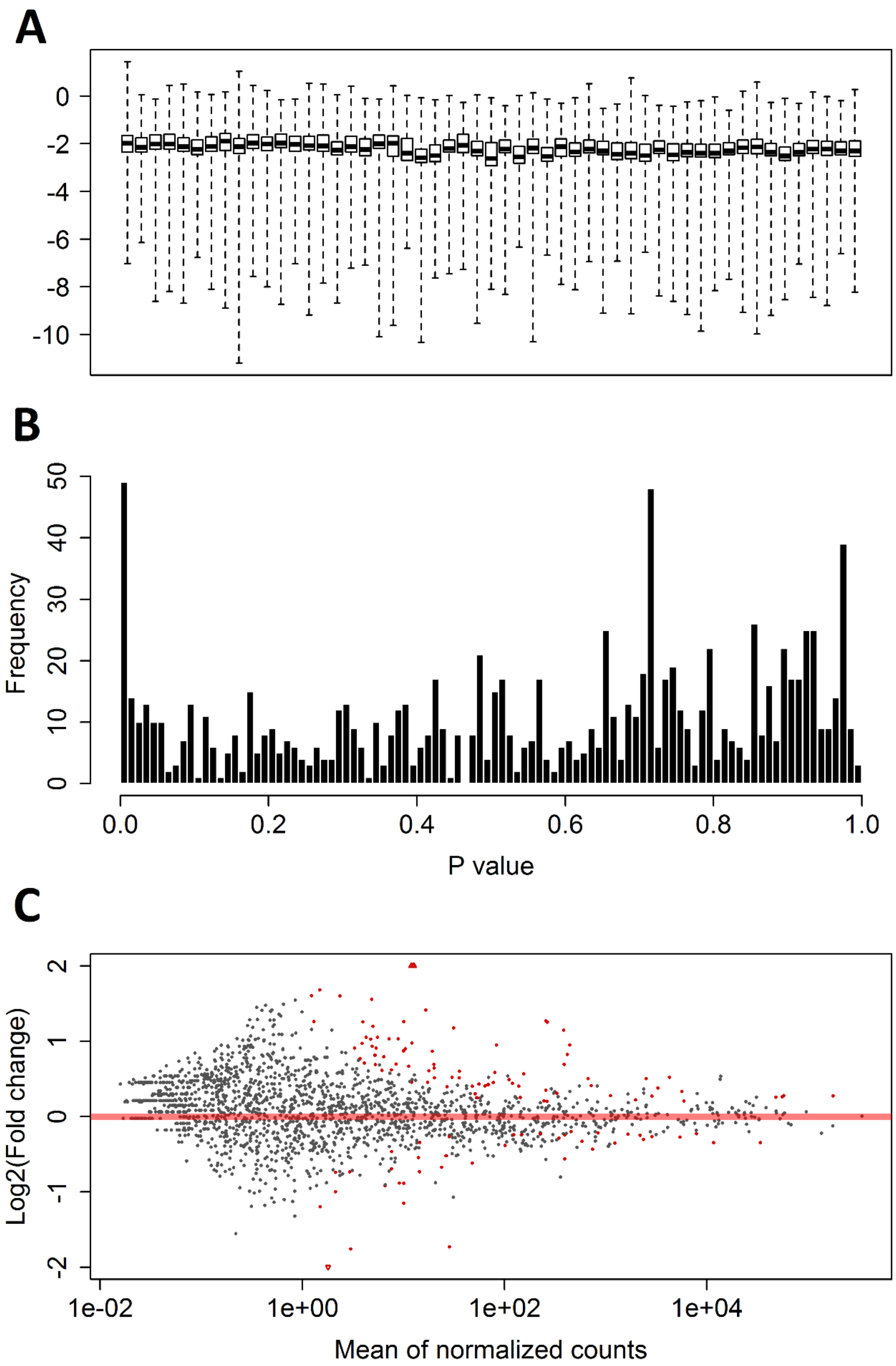

Figure S2. Control plots for miRNA sequencing data. **(A)** Boxplot of Cook's distances of miRNAs across samples. Whiskers cover the region between minimum and maximum values of Cook's distance, boxes include the region between 25% and 75% quartile, horizontal lines inside boxes mark median value. **(B)** Histogram of *P* values received from DESeq2 analysis. **(C)** MA plot of DESeq2 results (log2 fold changes of differentially expressed miRNAs over the average of normalized counts). Red points on MA plot indicate differentially expressed miRNAs with *P* values < 0.1.

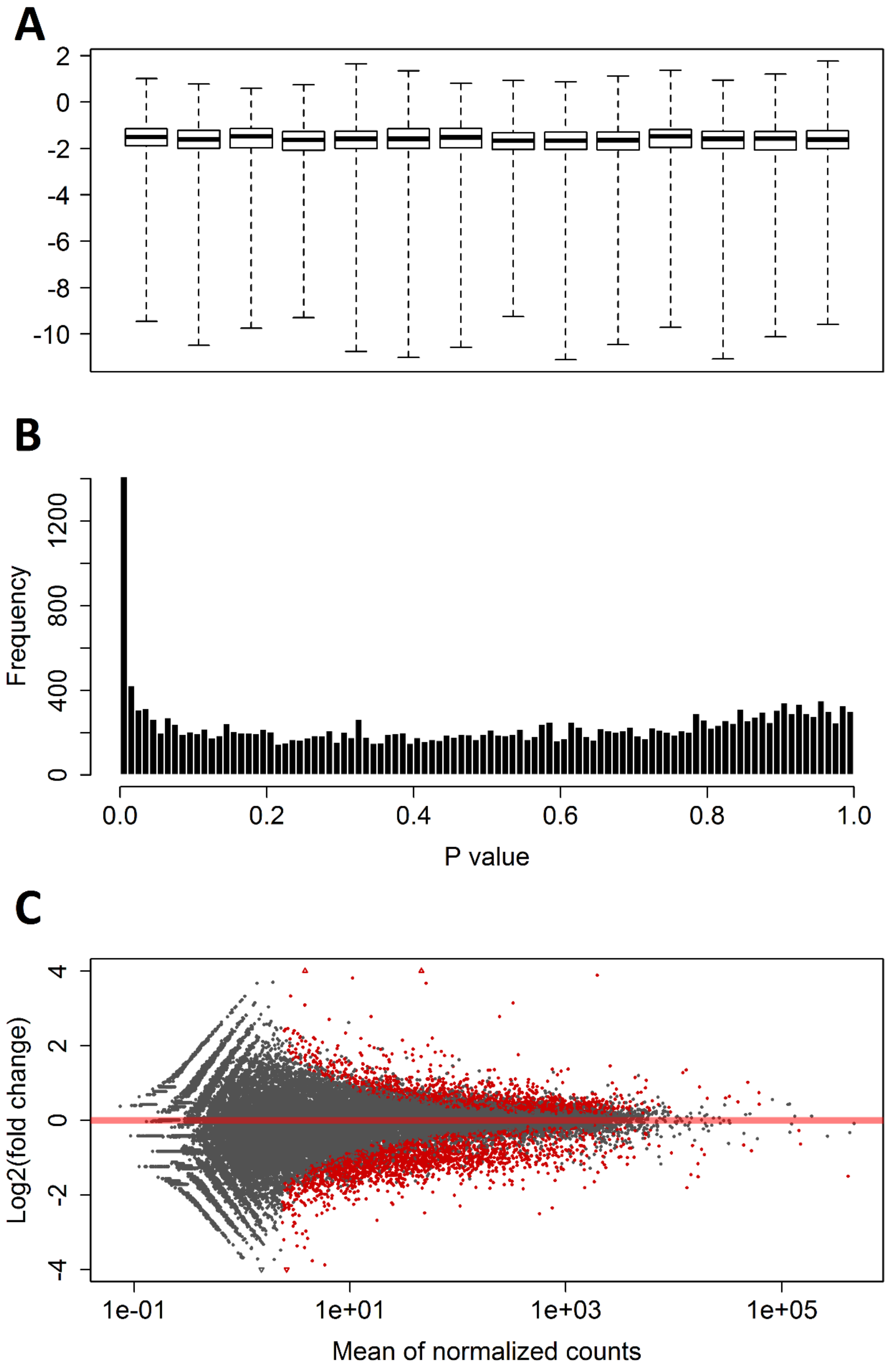

Figure S3. Control plots for transcriptome sequencing data. **(A)** Boxplot of Cook's distances of samples. Whiskers cover the region between minimum and maximum value of Cook's distance, boxes include the region between 25% and 75% quartile, horizontal lines inside boxes mark median value. **(B)** Histogram of *P* values received from DESeq2 analysis. **(C)** MA plot of DESeq2 results (log2 fold changes of differentially expressed genes over the average of normalized counts). Red points on MA plot indicate differentially expressed genes with *P* values < 0.1.

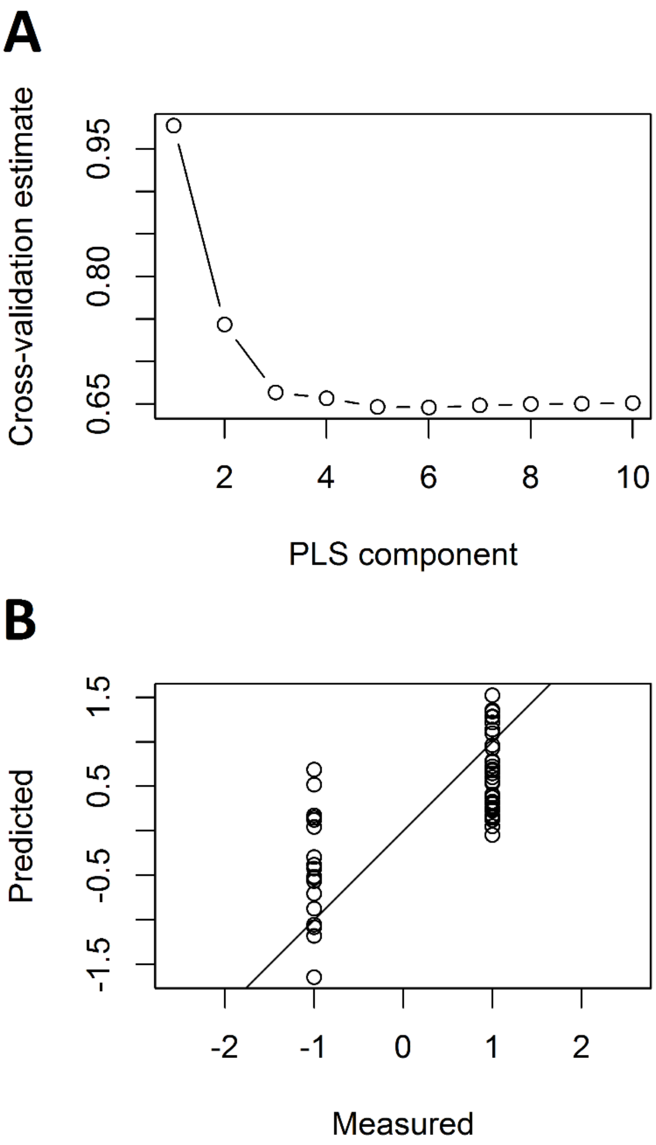

Figure S4. Plots for UVE-PLS analysis of miRNA expression in CVD group in comparison to control group. **(A)** The arrangement of prediction error and PLS components. **(B)** Plot of cross-validated predictions versus measured values.

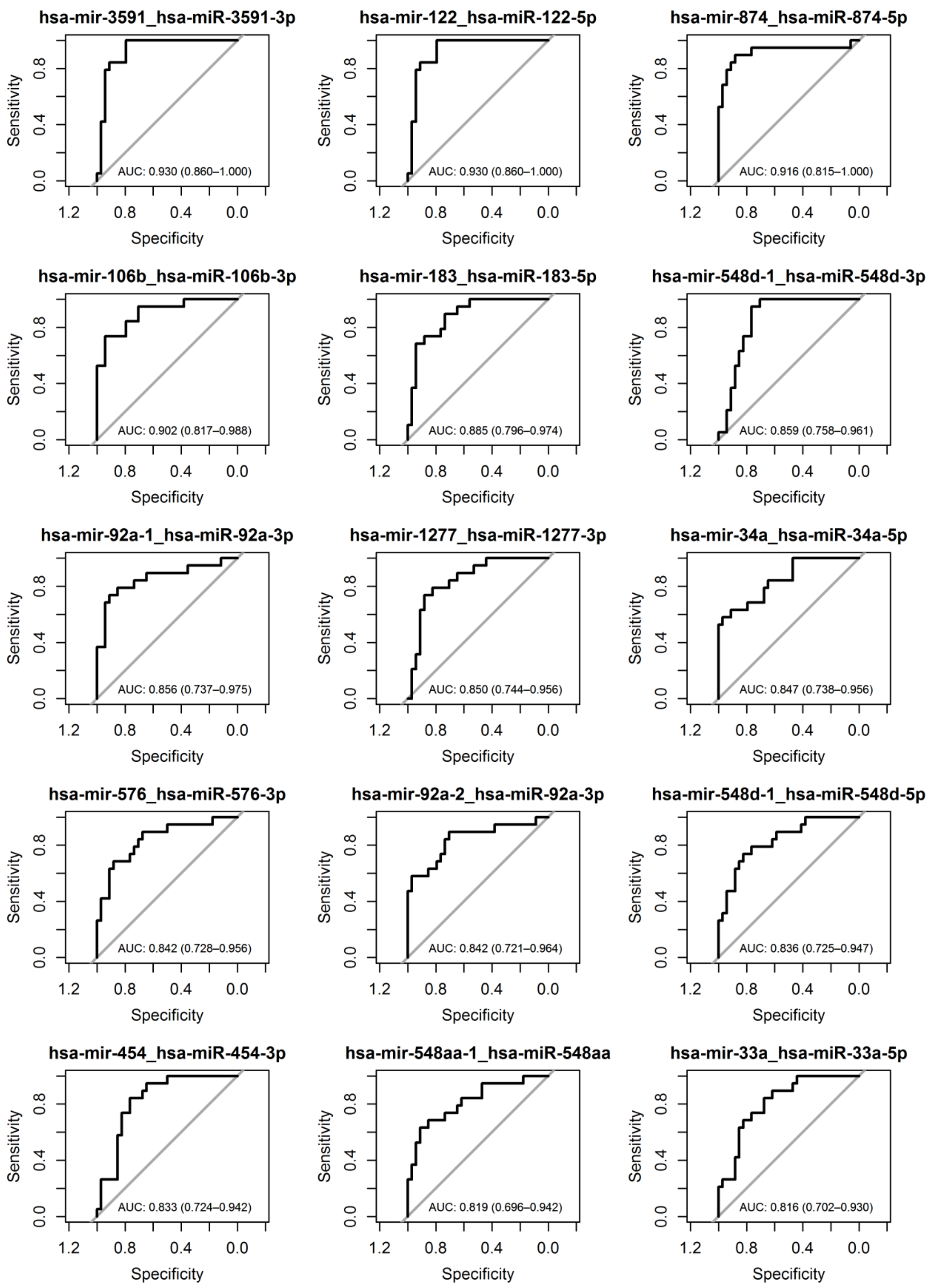

Figure S5. Receiver Operating Characteristics (ROC) curves for 34 miRNA transcripts selected as signatures of CVD. Plots include values of AUC (areas under curves) with 95% confidence interval in brackets.

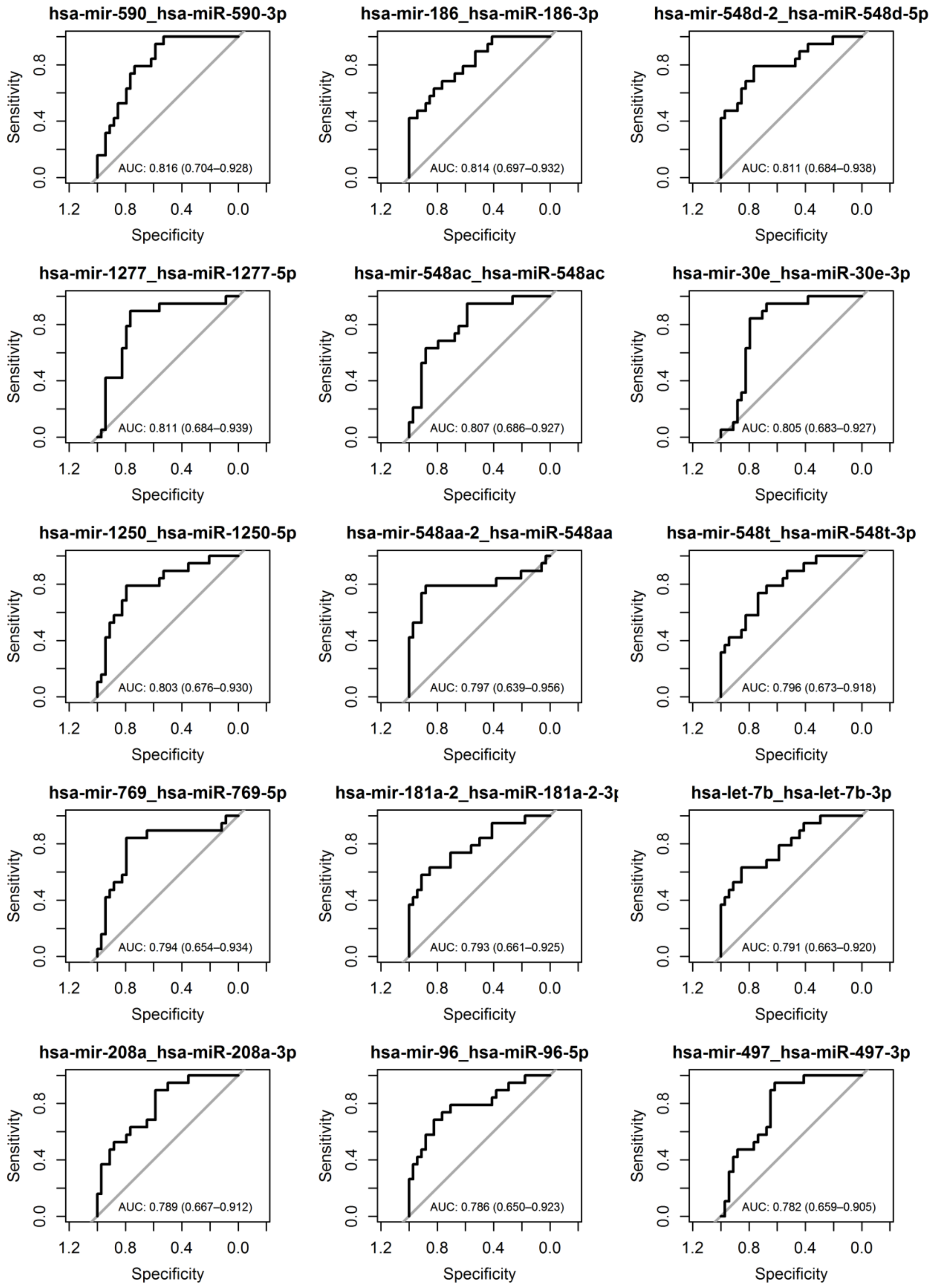

Figure S5. (continued).

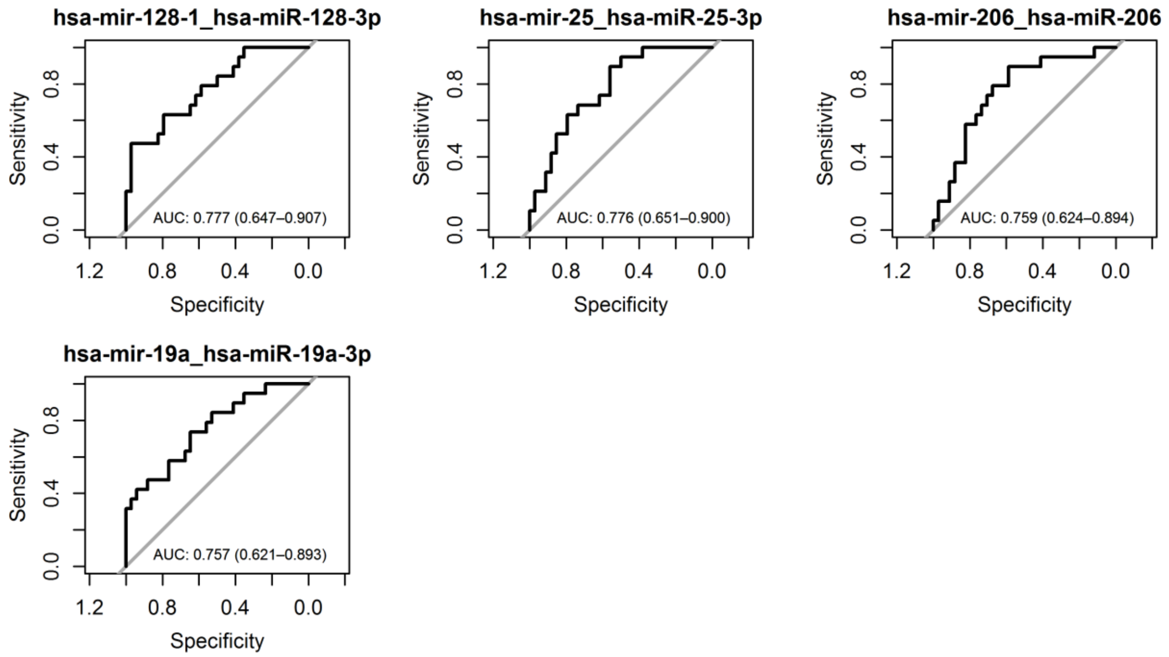

Figure S5. (continued).

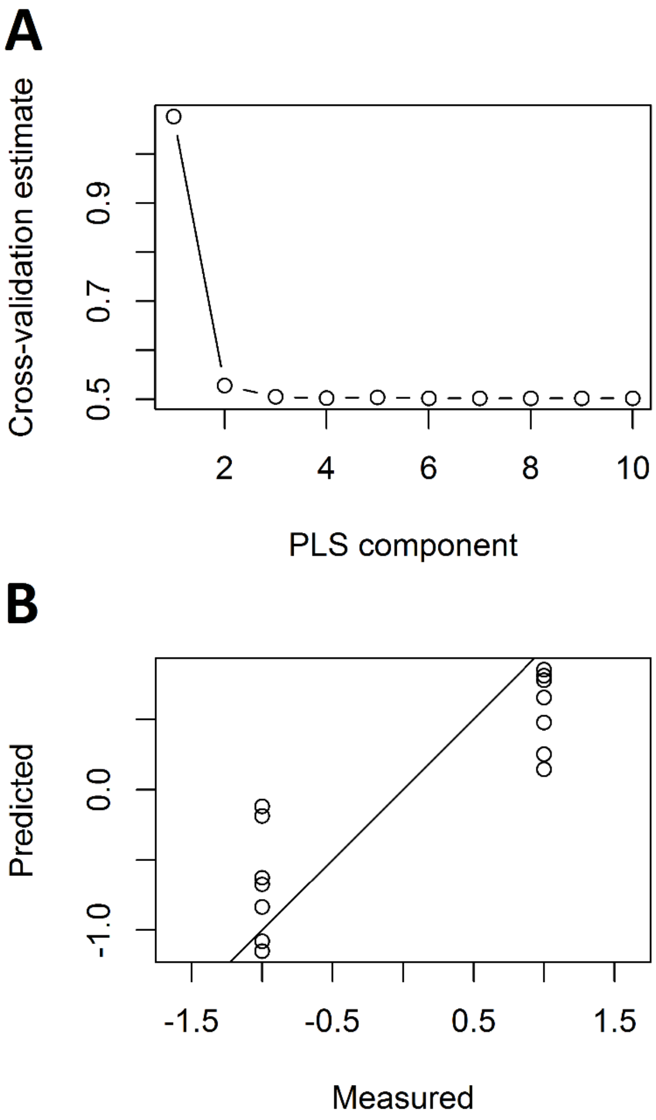

Figure S6. Plots for UVE-PLS analysis of genes expression in CVD group compared to control group. **(A)** The arrangement of prediction error and PLS components. **(B)** Plot of cross-validated predictions versus measured values.

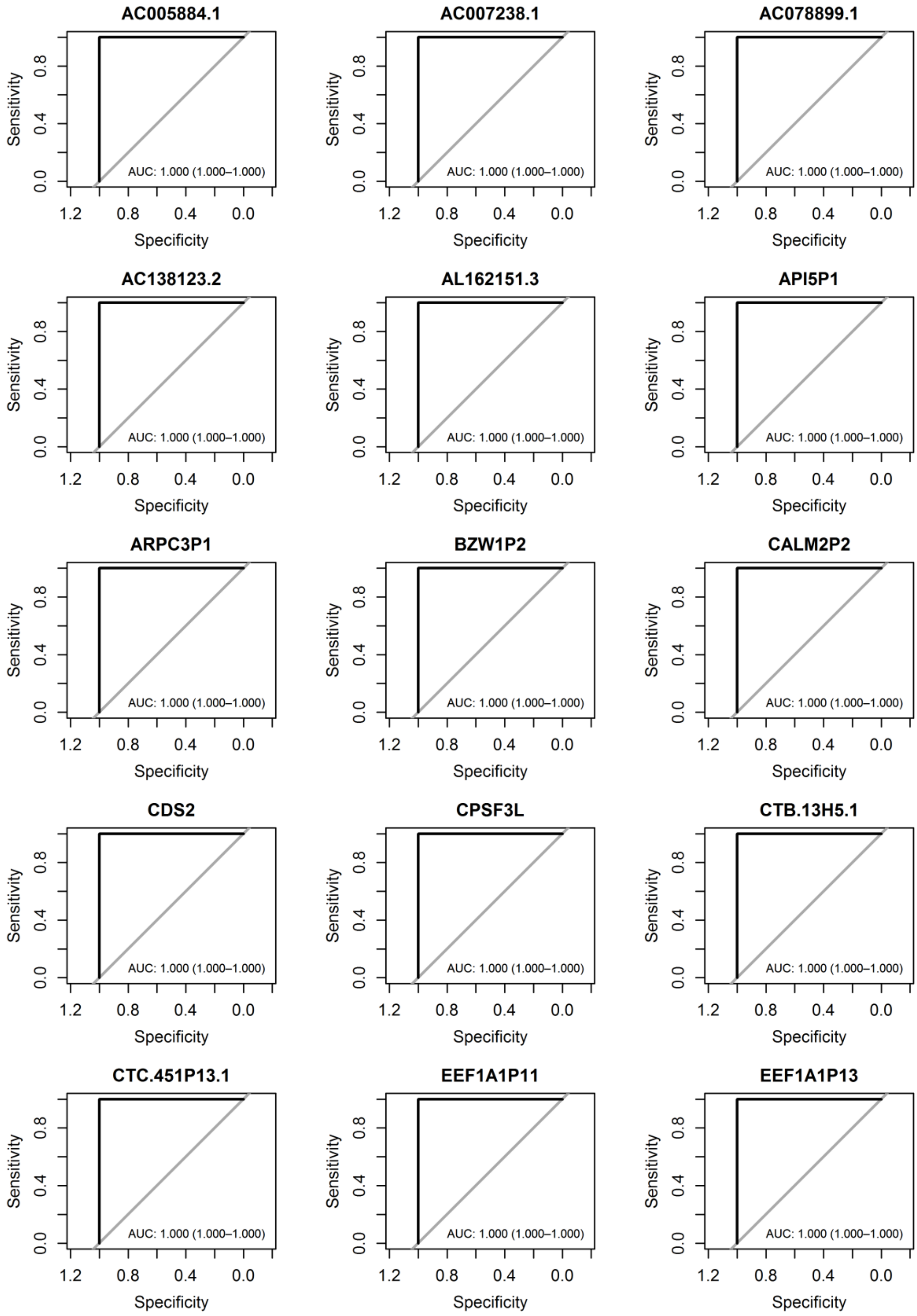

Figure S7. Receiver Operating Characteristics (ROC) curves for 62 genes selected as signatures of CVD. Plots include values of AUC (areas under curves) with 95% confidence interval in brackets.

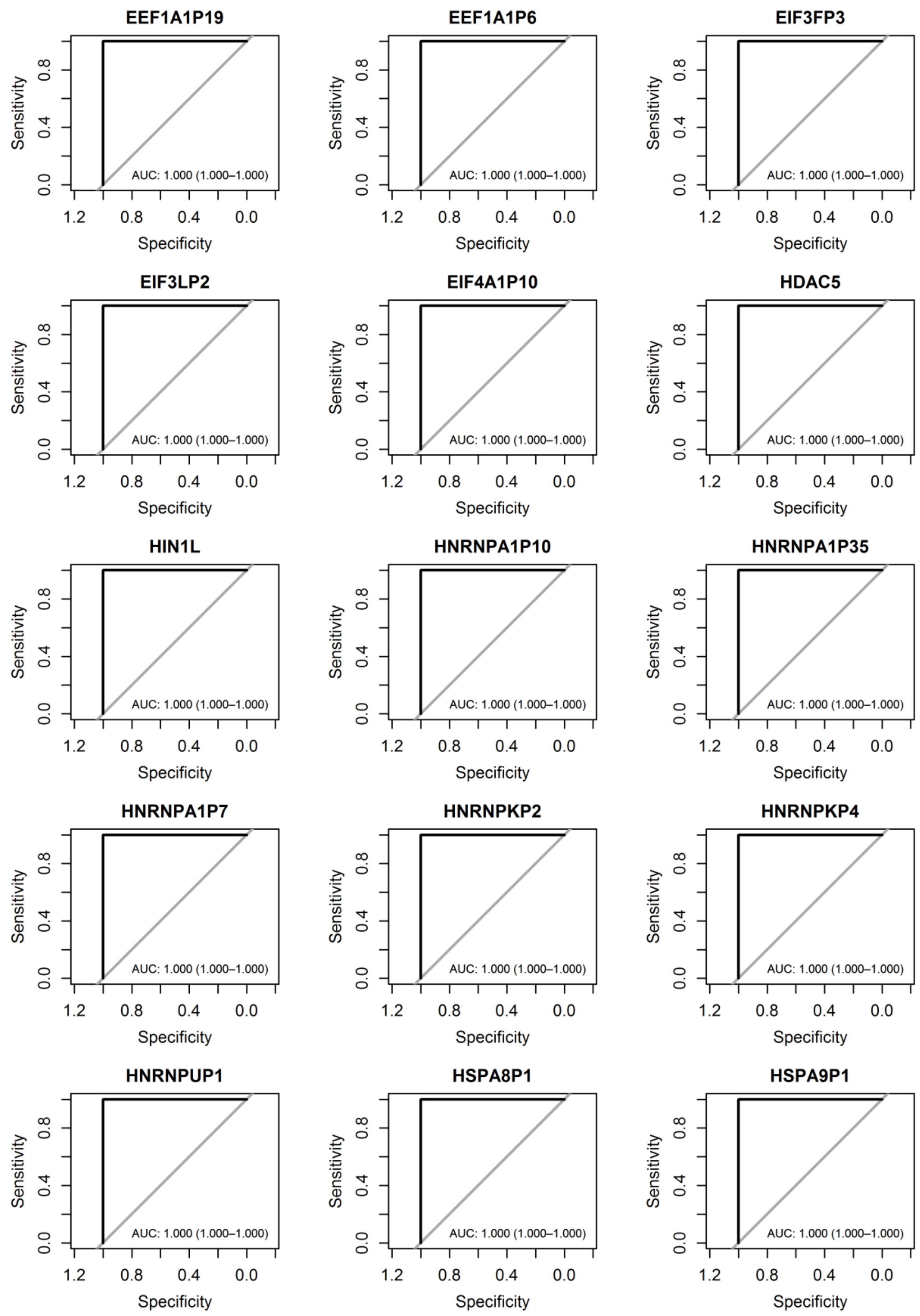

Figure S7. (continued).

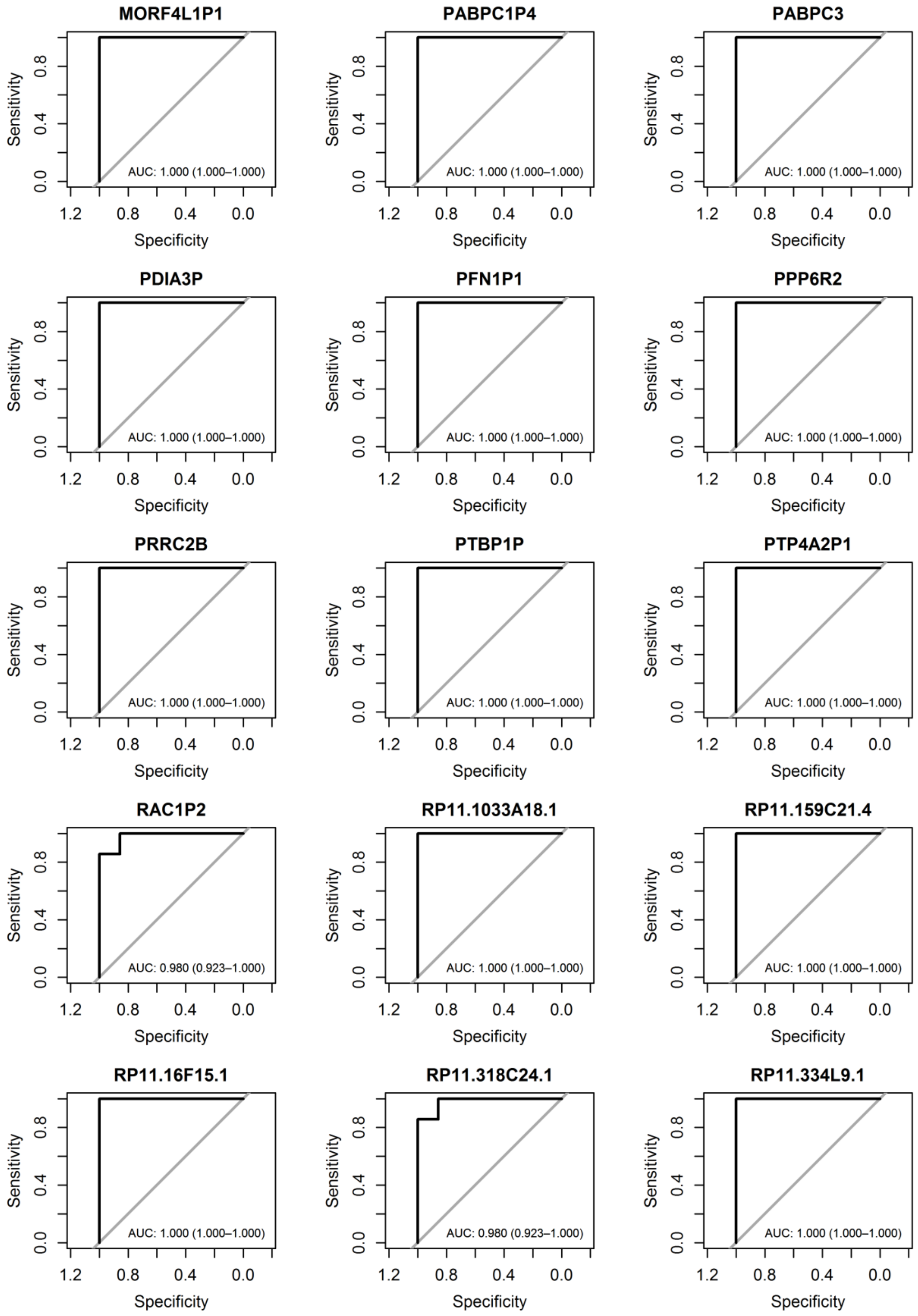

Figure S7. (continued).

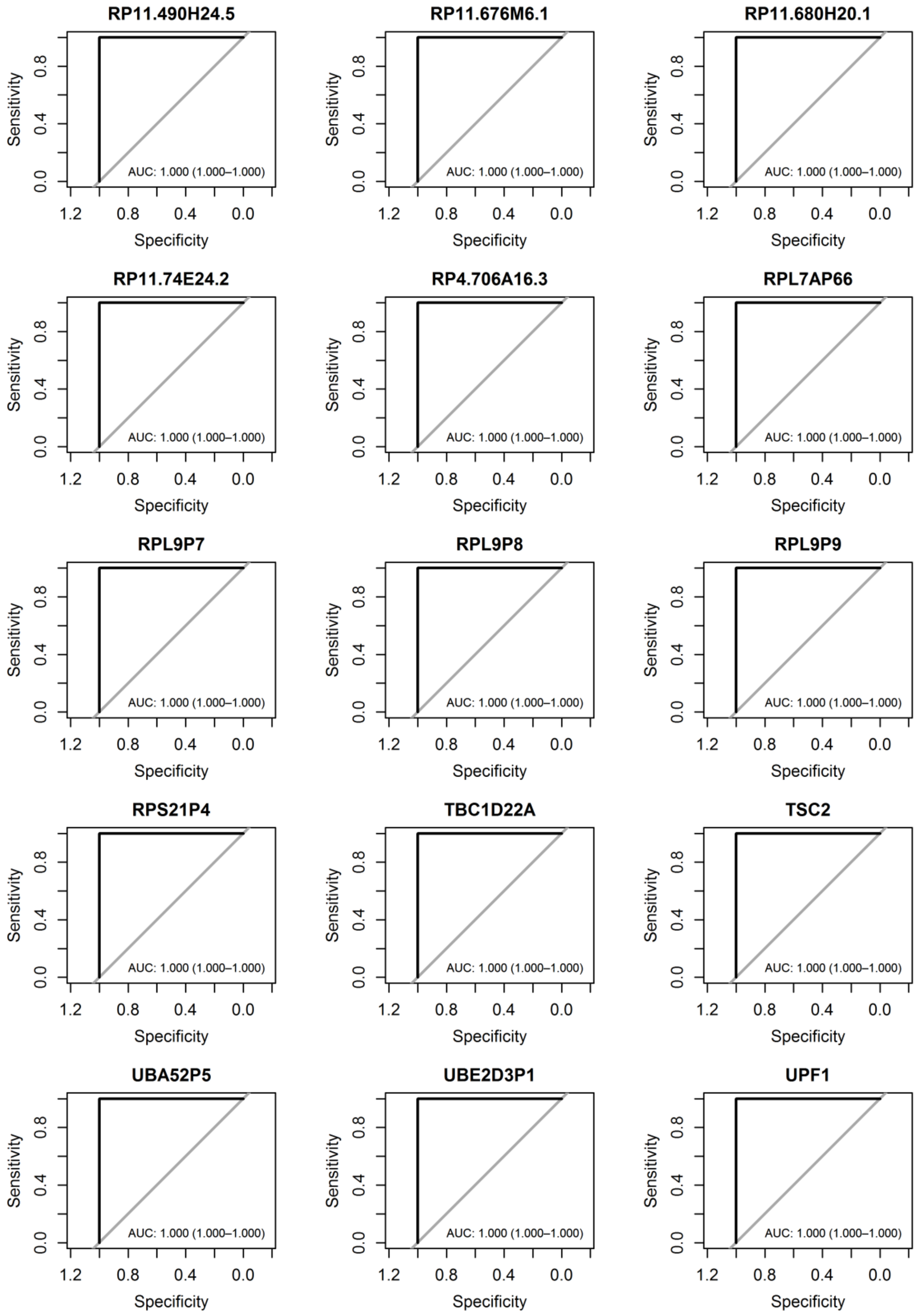

Figure S7. (continued).

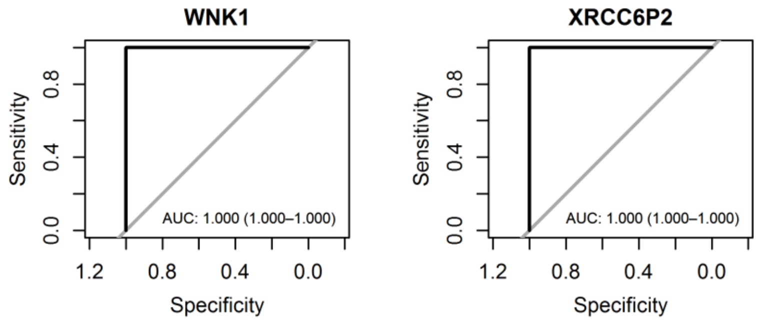

Figure S7. (continued).

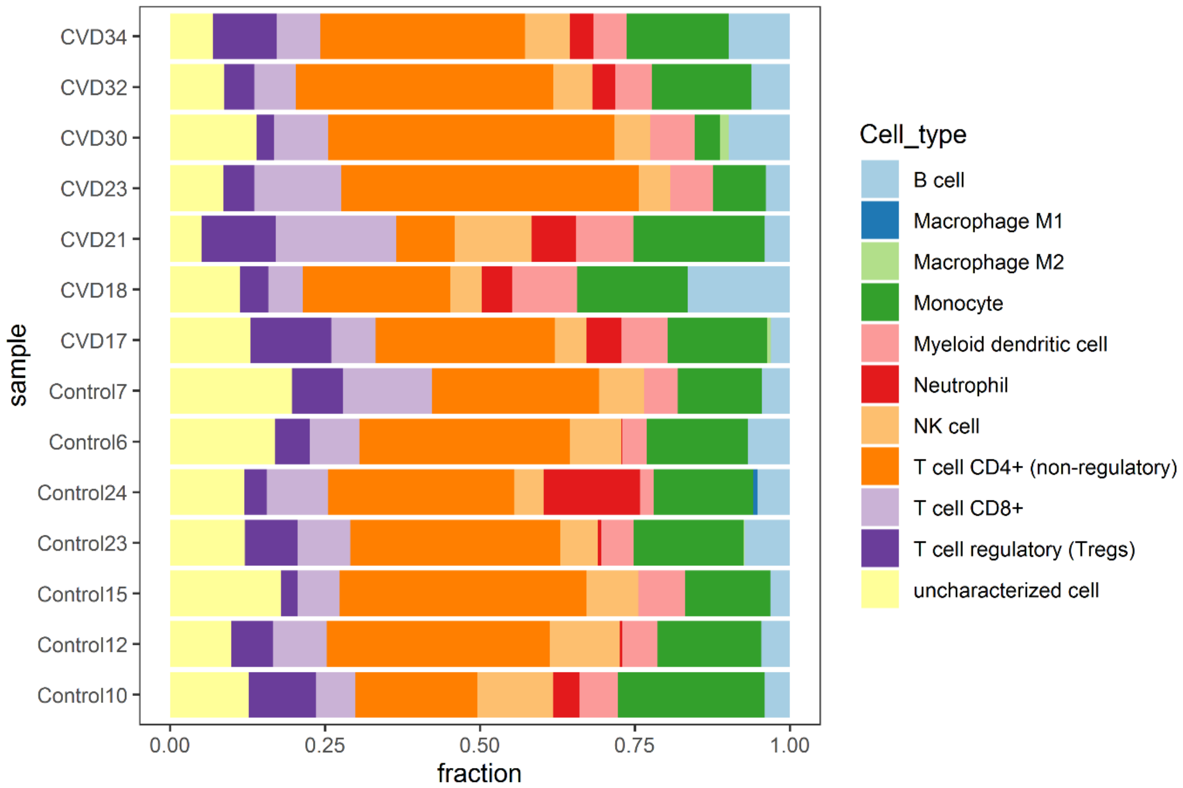

Figure S8. Results of gene expression deconvolution procedure for seven CVD patients and seven control subjects performed using "quanTIseq" method implemented to immunedeconv 2.0.0 package.

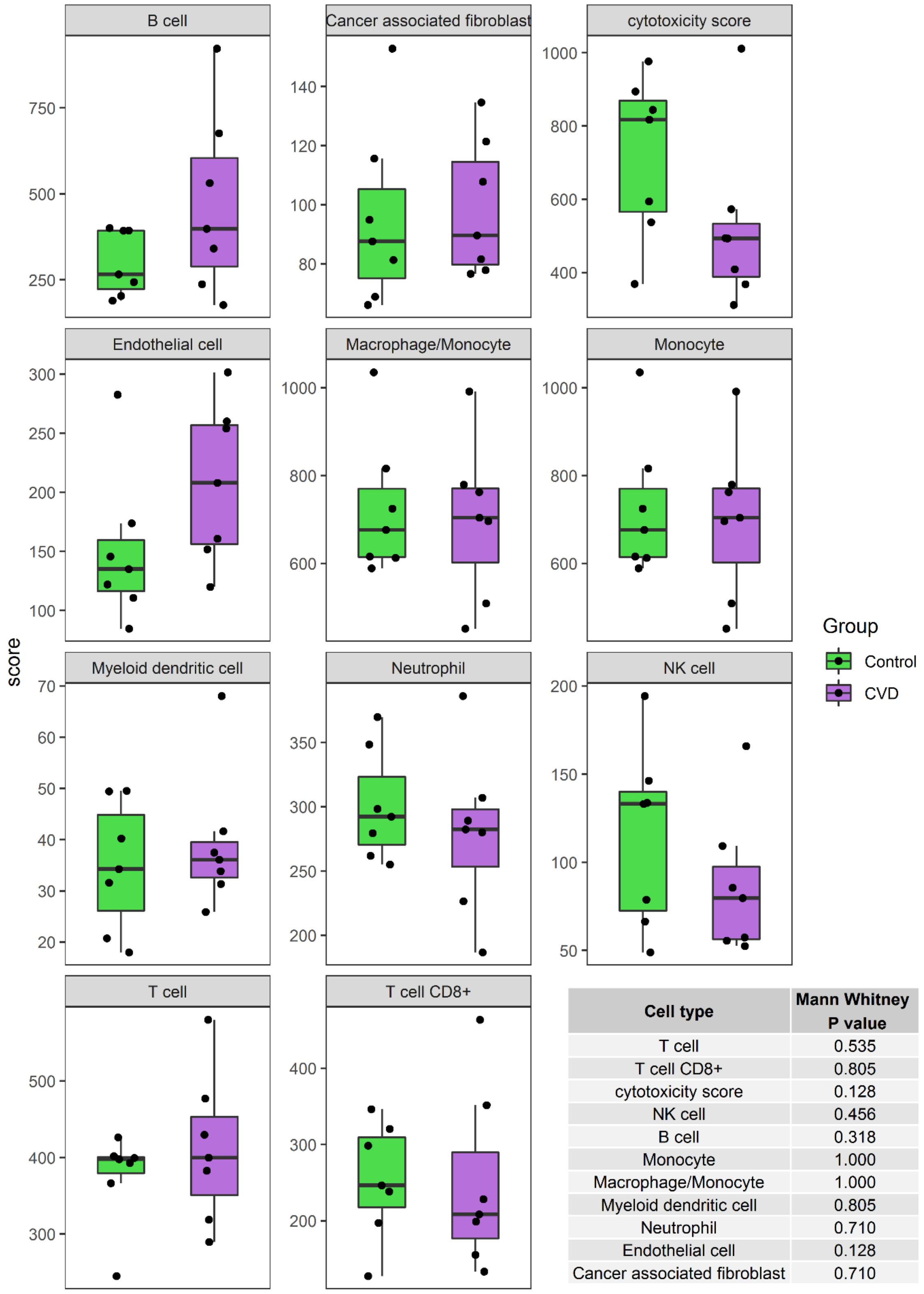

Cell type	Mann Whitney P value
T cell	0.535
T cell CD8+	0.805
cytotoxicity score	0.128
NK cell	0.456
B cell	0.318
Monocyte	1.000
Macrophage/Monocyte	1.000
Myeloid dendritic cell	0.805
Neutrophil	0.710
Endothelial cell	0.128
Cancer associated fibroblast	0.710

Figure S9. Results of deconvolution procedure performed on gene expression datasets of 7 CVD patients (CVD) and 7 control subjects (Control) using “MCPcounter” method implemented to immunedeconv 2.0.0 package. Statistical significance of differences in score values between CVD and control groups were calculated using two-sided Mann Whitney test (wilcox.test function in R).

Supplementary Tables

Table S1. Measurements of small RNA samples and small RNA libraries as well as results of small RNA sequencing data analysis received from Ion Torrent small RNA Plugin v5.0.5r3.

Parameter	Mean ± Standard Deviation	Median
Percentage of microRNA in small RNA samples	17.98 ± 8.14	18
Percentage of 94-114 bp* region in small RNA libraries	48.84 ± 6.27	49
Molar concentration of small RNA libraries (pM)	135,715.8 ± 42,658.12	137,815
Ion Sphere Particles enrichment quality control	31.02% ± 4.581%	30%
Total number of reads	8,779,221.98 ± 4,331,384.11	8,108,177
Reads passing filter	7,903,734.32 ± 4,055,551.56	7,525,029
Number of aligned reads	7,355,398.49 ± 3,814,853.06	6,990,778
Percentage of aligned reads	92.67% ± 2.19%	93.06%
Number of reads aligned to mirBase v21	2,800,864.9 ± 1,603,122.07	2,487,376
Percentage of reads aligned to mirBase v21	36.93% ± 8.06%	36.86%
Number of reads aligned to precursors	114,344.79 ± 62,594.61	104,094
microRNAs with 1+ reads	1,106.93 ± 301.57	1,173
microRNAs with 10+ reads	597.26 ± 87.07	599
microRNAs with 100+ reads	304.60 ± 53.05	306
microRNAs with 1,000+ reads	139.95 ± 26.13	139
microRNAs with 10,000+ reads	57.58 ± 17.97	61
Non-uniquely mapped reads	0	0
No feature mappings	495.46 ± 304.01	404
Ambiguous mappings	0	0
Percentage of coding reads	18.38% ± 3.22%	18.19%
Percentage of rRNA reads	0.81% ± 0.91%	0.55%
Percentage of tRNA reads	7.52% ± 6.63%	4.16%
Percentage of snoRNA reads	8.79% ± 3.68%	8.62%
Percentage of snRNA reads	0.93% ± 0.44%	0.9%
Percentage of lincRNA reads	1.19% ± 0.31%	1.21%
Percentage of pseudogene reads	0.28% ± 0.06%	0.27%

*base pair

Table S2. Measurements of transcriptome libraries and results of transcriptome sequencing data analysis received from Ion Torrent RNASeqAnalysis plugin v.5.0.3.0.

**Parameter**	**Mean ± Standard Deviation**	**Median**
Molar concentration of libraries (pM)	82,000 ± 22,577.69	76,950
Percentage of 50-160 bp* fragments in libraries	13.93 ± 6.75	13
Ion Sphere Particles enrichment quality control	23.429% ± 4.033%	21%
Total reads	35,630,503.93 ± 6,171,010.6	35,028,745
Aligned reads	34,137,367.79 ± 5,698,815.7	33,333,896
Percent aligned reads	95.92% ± 1.59%	96.31%
Mean read length	106.11 ± 15.461	104.05
Genes Detected	15,595.5 ± 4,062.45	16,405.5
Isoforms Detected	48,435.71 ± 5,951.03	47,947
Reads mapped to genes	10,798,201.43 ± 2,996,793.34	10,362,428
Genes with 1+ reads	30,568 ± 1,153.19	30,599
Genes with 10+ reads	15,595.5 ± 4,062.45	16,405.5
Genes with 100+ reads	8,387.4 ± 2,354.73	8,896.5
Genes with 1000+ reads	1,654.57 ± 587.45	1,545
Genes with 10000+ reads	77.5 ± 22.66	73.5
Total base reads	3,769,611,717 ± 732,961,248.3	3,787,703,121
Total aligned bases	3,090,546,914 ± 607,390,161	2,972,554,880
Percent aligned bases	82.21% ± 4.42%	83.37%
Percent coding bases	13.25% ± 5.06%	11.55%
Percent UTR† bases	25.63% ± 7.44%	22.88%
Percent ribosomal bases	5.37% ± 1.51%	5.23%
Percent intronic bases	40.22% ± 5.51%	41.22%
Percent intergenic bases	16.33% ± 6.47%	18.76%
Strand balance	50.24% ± 1.12%	50.32%

*base pair; †Untranslated Region

Table S3. The set of 96 differentially expressed microRNA transcripts resulted from DESeq2 analysis with $P < 0.05$ in 34 patients with CVD compared to 19 controls. Presented microRNA transcripts were ordered according to increasing P value.

No.	microRNA transcript	*P* value	Fold change
1.	hsa-mir-122_hsa-miR-122-5p	1.061E-09	2.213
2.	hsa-mir-3591_hsa-miR-3591-3p	1.061E-09	2.213
3.	hsa-mir-124-2_hsa-miR-124-3p	5.001E-07	16.991
4.	hsa-mir-183_hsa-miR-183-5p	2.052E-06	1.932
5.	hsa-mir-124-1_hsa-miR-124-3p	2.127E-05	10.053
6.	hsa-mir-548d-1_hsa-miR-548d-3p	2.127E-05	1.617
7.	hsa-mir-1277_hsa-miR-1277-3p	2.127E-05	1.773
8.	hsa-mir-34a_hsa-miR-34a-5p	3.809E-05	1.931
9.	hsa-mir-92a-1_hsa-miR-92a-3p	7.893E-05	0.832
10.	hsa-mir-874_hsa-miR-874-5p	1.286E-04	0.543
11.	hsa-mir-486_hsa-miR-486-5p	1.720E-04	2.411
12.	hsa-mir-486-2_hsa-miR-486-3p	1.720E-04	2.413
13.	hsa-mir-486_hsa-miR-486-3p	2.110E-04	2.384
14.	hsa-mir-486-2_hsa-miR-486-5p	2.110E-04	2.384
15.	hsa-mir-106b_hsa-miR-106b-3p	2.468E-04	0.796
16.	hsa-mir-454_hsa-miR-454-3p	3.039E-04	1.213
17.	hsa-mir-576_hsa-miR-576-3p	3.039E-04	2.043
18.	hsa-mir-92a-2_hsa-miR-92a-3p	3.039E-04	0.841
19.	hsa-mir-124-3_hsa-miR-124-3p	3.441E-04	8.268
20.	hsa-mir-548d-1_hsa-miR-548d-5p	3.441E-04	1.349
21.	hsa-mir-186_hsa-miR-186-3p	3.608E-04	1.357
22.	hsa-mir-548d-2_hsa-miR-548d-5p	3.608E-04	1.350
23.	hsa-mir-548aa-1_hsa-miR-548aa	5.130E-04	1.325
24.	hsa-mir-548aa-2_hsa-miR-548aa	1.020E-03	1.338
25.	hsa-mir-33a_hsa-miR-33a-5p	1.020E-03	1.207
26.	hsa-mir-590_hsa-miR-590-3p	1.020E-03	1.166
27.	hsa-mir-181a-2_hsa-miR-181a-2-3p	1.020E-03	0.677
28.	hsa-mir-548t_hsa-miR-548t-3p	1.808E-03	1.323
29.	hsa-mir-1277_hsa-miR-1277-5p	1.837E-03	1.329
30.	hsa-let-7b_hsa-let-7b-3p	2.056E-03	1.322
31.	hsa-mir-548e_hsa-miR-548e-3p	2.502E-03	1.429
32.	hsa-mir-3960_hsa-miR-3960	2.533E-03	0.451
33.	hsa-mir-128-1_hsa-miR-128-3p	2.667E-03	0.850
34.	hsa-mir-4284_hsa-miR-4284	2.709E-03	0.302
35.	hsa-mir-96_hsa-miR-96-5p	3.726E-03	2.291
36.	hsa-mir-22_hsa-miR-22-3p	4.626E-03	1.259
37.	hsa-mir-30e_hsa-miR-30e-3p	5.532E-03	0.740
38.	hsa-mir-548ac_hsa-miR-548ac	5.532E-03	1.761
39.	hsa-mir-664a_hsa-miR-664a-5p	5.532E-03	1.315
40.	hsa-mir-769_hsa-miR-769-5p	5.532E-03	0.871
41.	hsa-mir-19a_hsa-miR-19a-3p	5.824E-03	1.194
42.	hsa-mir-506_hsa-miR-506-5p	6.776E-03	2.388
43.	hsa-mir-206_hsa-miR-206	7.995E-03	2.036
44.	hsa-mir-1250_hsa-miR-1250-5p	8.559E-03	0.619
45.	hsa-mir-196a-2_hsa-miR-196a-5p	8.922E-03	2.663

46.	hsa-mir-7641-1_hsa-miR-7641	8.944E-03	2.263
47.	hsa-mir-25_hsa-miR-25-3p	8.944E-03	0.860
48.	hsa-mir-497_hsa-miR-497-3p	9.309E-03	1.437
49.	hsa-mir-208a_hsa-miR-208a-3p	9.808E-03	3.208
50.	hsa-mir-497_hsa-miR-497-5p	1.165E-02	1.480
51.	hsa-mir-548d-2_hsa-miR-548d-3p	1.165E-02	1.429
52.	hsa-mir-548f-2_hsa-miR-548f-3p	1.165E-02	1.755
53.	hsa-mir-424_hsa-miR-424-3p	1.210E-02	1.500
54.	hsa-mir-424_hsa-miR-424-5p	1.541E-02	1.418
55.	hsa-mir-454_hsa-miR-454-5p	1.566E-02	1.232
56.	hsa-mir-330_hsa-miR-330-3p	1.666E-02	0.849
57.	hsa-mir-339_hsa-miR-339-3p	1.677E-02	0.846
58.	hsa-mir-196a-1_hsa-miR-196a-5p	1.697E-02	2.393
59.	hsa-mir-7847_hsa-miR-7847-3p	1.711E-02	1.909
60.	hsa-mir-6836_hsa-miR-6836-3p	1.711E-02	0.296
61.	hsa-mir-450b_hsa-miR-450b-5p	1.711E-02	1.520
62.	hsa-mir-5582_hsa-miR-5582-3p	1.921E-02	1.878
63.	hsa-mir-503_hsa-miR-503-5p	1.921E-02	1.419
64.	hsa-mir-4433b_hsa-miR-4433b-3p	2.015E-02	1.617
65.	hsa-mir-4433_hsa-miR-4433-5p	2.015E-02	1.617
66.	hsa-mir-598_hsa-miR-598-3p	2.081E-02	1.154
67.	hsa-mir-542_hsa-miR-542-3p	2.228E-02	1.405
68.	hsa-let-7f-1_hsa-let-7f-1-3p	2.343E-02	1.193
69.	hsa-mir-19b-1_hsa-miR-19b-3p	2.563E-02	1.212
70.	hsa-mir-1246_hsa-miR-1246	2.563E-02	3.038
71.	hsa-mir-34a_hsa-miR-34a-3p	2.707E-02	1.863
72.	hsa-mir-4423_hsa-miR-4423-3p	2.788E-02	2.942
73.	hsa-mir-335_hsa-miR-335-5p	2.788E-02	1.436
74.	hsa-mir-19b-2_hsa-miR-19b-3p	3.123E-02	1.199
75.	hsa-mir-490_hsa-miR-490-3p	3.227E-02	2.041
76.	hsa-mir-146b_hsa-miR-146b-5p	3.228E-02	0.786
77.	hsa-mir-340_hsa-miR-340-5p	3.372E-02	1.156
78.	hsa-mir-135a-1_hsa-miR-135a-5p	3.377E-02	1.561
79.	hsa-mir-3198-2_hsa-miR-3198	3.456E-02	1.959
80.	hsa-mir-34b_hsa-miR-34b-5p	3.462E-02	1.734
81.	hsa-mir-138-2_hsa-miR-138-5p	3.468E-02	0.601
82.	hsa-mir-195_hsa-miR-195-5p	3.468E-02	1.238
83.	hsa-mir-599_hsa-miR-599	3.511E-02	0.436
84.	hsa-mir-193b_hsa-miR-193b-3p	3.547E-02	0.651
85.	hsa-mir-4664_hsa-miR-4664-5p	3.559E-02	0.601
86.	hsa-mir-181a-2_hsa-miR-181a-5p	3.683E-02	0.854
87.	hsa-mir-222_hsa-miR-222-3p	4.029E-02	0.828
88.	hsa-mir-619_hsa-miR-619-5p	4.324E-02	0.541
89.	hsa-mir-138-1_hsa-miR-138-5p	4.615E-02	0.627
90.	hsa-mir-885_hsa-miR-885-5p	4.812E-02	2.074
91.	hsa-mir-200b_hsa-miR-200b-5p	4.819E-02	1.830
92.	hsa-mir-186_hsa-miR-186-5p	4.819E-02	1.299
93.	hsa-mir-342_hsa-miR-342-3p	4.819E-02	0.786
94.	hsa-mir-583_hsa-miR-583	4.819E-02	2.072
95.	hsa-mir-1268b_hsa-miR-1268b	4.913E-02	1.590
96.	hsa-mir-21_hsa-miR-21-5p	4.982E-02	1.209

Table S4. The set of 48 differentially expressed microRNA transcripts resulted from UVE-PLS analysis in in 34 patients with CVD group compared to 19 controls. Presented microRNA transcripts were ordered according to decreasing PLS coefficients.

No	microRNA transcript	PLS coefficient
1.	hsa-mir-122_hsa-miR-122-5p	4.713E-02
2.	hsa-mir-3591_hsa-miR-3591-3p	4.706E-02
3.	hsa-mir-1277_hsa-miR-1277-3p	4.037E-02
4.	hsa-mir-296_hsa-miR-296-5p	4.009E-02
5.	hsa-mir-183_hsa-miR-183-5p	3.828E-02
6.	hsa-mir-34a_hsa-miR-34a-5p	3.447E-02
7.	hsa-mir-576_hsa-miR-576-3p	3.210E-02
8.	hsa-mir-548ac_hsa-miR-548ac	2.868E-02
9.	hsa-mir-208a_hsa-miR-208a-3p	2.770E-02
10.	hsa-mir-206_hsa-miR-206	2.760E-02
11.	hsa-mir-96_hsa-miR-96-5p	2.644E-02
12.	hsa-mir-1277_hsa-miR-1277-5p	2.130E-02
13.	hsa-mir-548d-1_hsa-miR-548d-3p	2.093E-02
14.	hsa-mir-335_hsa-miR-335-5p	1.940E-02
15.	hsa-mir-186_hsa-miR-186-3p	1.651E-02
16.	hsa-mir-497_hsa-miR-497-3p	1.629E-02
17.	hsa-mir-548d-1_hsa-miR-548d-5p	1.470E-02
18.	hsa-mir-548d-2_hsa-miR-548d-5p	1.465E-02
19.	hsa-mir-548aa-1_hsa-miR-548aa	1.459E-02
20.	hsa-mir-548aa-2_hsa-miR-548aa	1.456E-02
21.	hsa-mir-454_hsa-miR-454-5p	1.453E-02
22.	hsa-mir-19b-1_hsa-miR-19b-3p	1.217E-02
23.	hsa-mir-33a_hsa-miR-33a-5p	1.130E-02
24.	hsa-let-7b_hsa-let-7b-3p	1.091E-02
25.	hsa-mir-454_hsa-miR-454-3p	1.050E-02
26.	hsa-mir-19a_hsa-miR-19a-3p	8.377E-03
27.	hsa-mir-548t_hsa-miR-548t-3p	8.103E-03
28.	hsa-mir-590_hsa-miR-590-3p	6.737E-03
29.	hsa-mir-128-1_hsa-miR-128-3p	-7.843E-03
30.	hsa-mir-25_hsa-miR-25-3p	-8.997E-03
31.	hsa-mir-342_hsa-miR-342-3p	-1.069E-02
32.	hsa-mir-769_hsa-miR-769-5p	-1.146E-02
33.	hsa-mir-106b_hsa-miR-106b-3p	-1.146E-02
34.	hsa-let-7g_hsa-let-7g-3p	-1.155E-02
35.	hsa-mir-330_hsa-miR-330-3p	-1.217E-02
36.	hsa-mir-378a_hsa-miR-378a-5p	-1.269E-02
37.	hsa-mir-92a-1_hsa-miR-92a-3p	-1.403E-02
38.	hsa-mir-378c_hsa-miR-378c	-1.412E-02
39.	hsa-mir-92a-2_hsa-miR-92a-3p	-1.433E-02
40.	hsa-mir-30e_hsa-miR-30e-3p	-1.507E-02
41.	hsa-mir-1301_hsa-miR-1301-3p	-1.530E-02
42.	hsa-mir-181b-1_hsa-miR-181b-5p	-1.719E-02
43.	hsa-mir-485_hsa-miR-485-3p	-1.937E-02
44.	hsa-mir-181b-2_hsa-miR-181b-5p	-2.242E-02

45.	hsa-mir-193b_hsa-miR-193b-3p	-2.251E-02
46.	hsa-mir-181a-2_hsa-miR-181a-2-3p	-3.243E-02
47.	hsa-mir-1250_hsa-miR-1250-5p	-3.325E-02
48.	hsa-mir-874_hsa-miR-874-5p	-3.432E-02

Table S5. Results of ROC analysis for 34 miRNA transcripts selected as indicative for CVD.

miRNA transcript	ROC-AUC[1]	Threshold	Specificity	Sensitivity	Accuracy	Positive Predictive Value	Negative Predictive Value
hsa-mir-122_hsa-miR-122-5p	0.930	8.370	1.000	0.794	0.868	1.000	0.731
hsa-mir-3591_hsa-miR-3591-3p	0.930	8.370	1.000	0.794	0.868	1.000	0.731
hsa-mir-874_hsa-miR-874-5p	0.916	3.231	0.895	0.882	0.887	0.938	0.810
hsa-mir-106b_hsa-miR-106b-3p	0.902	9.288	0.737	0.941	0.868	0.865	0.875
hsa-mir-183_hsa-miR-183-5p	0.885	6.209	0.895	0.735	0.792	0.926	0.654
hsa-mir-548d-1_hsa-miR-548d-3p	0.859	4.268	0.947	0.765	0.830	0.963	0.692
hsa-mir-92a-1_hsa-miR-92a-3p	0.856	11.531	0.737	0.912	0.849	0.861	0.824
hsa-mir-1277_hsa-miR-1277-3p	0.850	8.298	0.737	0.882	0.830	0.857	0.778
hsa-mir-34a_hsa-miR-34a-5p	0.847	8.177	0.579	0.971	0.830	0.805	0.917
hsa-mir-576_hsa-miR-576-3p	0.842	3.131	0.895	0.676	0.755	0.920	0.607
hsa-mir-92a-2_hsa-miR-92a-3p	0.842	11.078	0.895	0.706	0.774	0.923	0.630
hsa-mir-548d-1_hsa-miR-548d-5p	0.836	5.703	0.737	0.824	0.792	0.848	0.700
hsa-mir-454_hsa-miR-454-3p	0.833	10.038	0.842	0.765	0.792	0.897	0.667
hsa-mir-548aa-1_hsa-miR-548aa	0.819	5.833	0.632	0.912	0.811	0.816	0.800
hsa-mir-33a_hsa-miR-33a-5p	0.816	11.459	0.842	0.676	0.736	0.885	0.593
hsa-mir-590_hsa-miR-590-3p	0.816	10.945	0.947	0.588	0.717	0.952	0.563
hsa-mir-186_hsa-miR-186-3p	0.814	6.794	0.632	0.824	0.755	0.800	0.667
hsa-mir-548d-2_hsa-miR-548d-5p	0.811	5.736	0.789	0.765	0.774	0.867	0.652
hsa-mir-1277_hsa-miR-1277-5p	0.811	9.430	0.895	0.765	0.811	0.929	0.680
hsa-mir-548ac_hsa-miR-548ac	0.807	2.365	0.947	0.588	0.717	0.952	0.563
hsa-mir-30e_hsa-miR-30e-3p	0.805	9.635	0.842	0.794	0.811	0.900	0.696
hsa-mir-1250_hsa-miR-1250-5p	0.803	3.005	0.789	0.794	0.792	0.871	0.682
hsa-mir-548aa-2_hsa-miR-548aa	0.797	5.956	0.789	0.882	0.849	0.882	0.789
hsa-mir-548t_hsa-miR-548t-3p	0.796	5.506	0.737	0.735	0.736	0.833	0.609
hsa-mir-769_hsa-miR-769-5p	0.794	8.815	0.842	0.794	0.811	0.900	0.696
hsa-mir-181a-2_hsa-miR-181a-2-3p	0.793	8.788	0.579	0.912	0.792	0.795	0.786
hsa-let-7b_hsa-let-7b-3p	0.791	6.923	0.632	0.853	0.774	0.806	0.706
hsa-mir-208a_hsa-miR-208a-3p	0.789	0.402	0.895	0.588	0.698	0.909	0.548
hsa-mir-96_hsa-miR-96-5p	0.786	1.916	0.684	0.824	0.774	0.824	0.684
hsa-mir-497_hsa-miR-497-3p	0.782	4.930	0.947	0.618	0.736	0.955	0.581
hsa-mir-128-1_hsa-miR-128-3p	0.777	10.835	0.474	0.971	0.792	0.767	0.900
hsa-mir-25_hsa-miR-25-3p	0.776	9.665	0.895	0.559	0.679	0.905	0.531
hsa-mir-206_hsa-miR-206	0.759	2.125	0.895	0.588	0.698	0.909	0.548
hsa-mir-19a_hsa-miR-19a-3p	0.757	15.562	0.737	0.647	0.679	0.815	0.538

[1]Area under ROC curve.

Table S6: Correlation analysis between age and expression of 34 selected miRNA transcripts in CVD group. MiRNA transcripts were ordered according to increasing *P* value.

miRNA transcript	Correlation coefficient	*P* value
hsa-mir-548ac_hsa-miR-548ac	-0.35	0.039
hsa-mir-1277_hsa-miR-1277-3p	0.31	0.077
hsa-mir-208a_hsa-miR-208a-3p	-0.30	0.084
hsa-mir-128-1_hsa-miR-128-3p	-0.26	0.133
hsa-mir-548d-1_hsa-miR-548d-3p	0.25	0.157
hsa-mir-19a_hsa-miR-19a-3p	-0.24	0.168
hsa-mir-92a-1_hsa-miR-92a-3p	-0.22	0.205
hsa-mir-186_hsa-miR-186-3p	-0.21	0.224
hsa-mir-576_hsa-miR-576-3p	-0.20	0.247
hsa-mir-874_hsa-miR-874-5p	0.20	0.252
hsa-mir-769_hsa-miR-769-5p	0.18	0.296
hsa-let-7b_hsa-let-7b-3p	0.16	0.364
hsa-mir-183_hsa-miR-183-5p	-0.15	0.395
hsa-mir-96_hsa-miR-96-5p	0.14	0.441
hsa-mir-206_hsa-miR-206	0.13	0.456
hsa-mir-92a-2_hsa-miR-92a-3p	-0.12	0.485
hsa-mir-30e_hsa-miR-30e-3p	-0.12	0.504
hsa-mir-34a_hsa-miR-34a-5p	0.12	0.512
hsa-mir-122_hsa-miR-122-5p	-0.12	0.514
hsa-mir-3591_hsa-miR-3591-3p	-0.12	0.515
hsa-mir-548t_hsa-miR-548t-3p	0.10	0.565
hsa-mir-497_hsa-miR-497-3p	0.10	0.576
hsa-mir-33a_hsa-miR-33a-5p	0.09	0.609
hsa-mir-1250_hsa-miR-1250-5p	-0.09	0.620
hsa-mir-25_hsa-miR-25-3p	0.09	0.627
hsa-mir-590_hsa-miR-590-3p	0.09	0.629
hsa-mir-548aa-2_hsa-miR-548aa	0.07	0.674
hsa-mir-548d-1_hsa-miR-548d-5p	0.06	0.721
hsa-mir-1277_hsa-miR-1277-5p	-0.05	0.757
hsa-mir-454_hsa-miR-454-3p	0.05	0.795
hsa-mir-548aa-1_hsa-miR-548aa	0.03	0.854
hsa-mir-548d-2_hsa-miR-548d-5p	0.03	0.859
hsa-mir-106b_hsa-miR-106b-3p	-0.02	0.922
hsa-mir-181a-2_hsa-miR-181a-2-3p	0.00	0.978

Table S7. 183 differentially expressed genes resulted from DESeq2 analysis with $P <$ 0.00001 in seven patients with CVD, compared to seven controls. Presented genes were ordered according to increasing *P* value. Gene names not assigned to gene symbols by HUGO Multi-symbol checker were termed as "Unmatched". Gene symbols in brackets are synonyms or previous gene symbols.

No.	Gene symbol	Gene name	*P* value	Fold change
1.	*TSC2*	TSC complex subunit 2	4.87E-17	1.4369
2.	*AC078899.1*	unmatched	1.18E-13	0.3926
3.	*RP11-16F15.1*	Unmatched	1.18E-13	0.3271
4.	*EEF1A1P19*	eukaryotic translation elongation factor 1 alpha 1 pseudogene 19	8.40E-13	0.4999
5.	*HSP90B2P*	heat shock protein 90 beta family member 2, pseudogene	4.04E-11	0.4115
6.	*PFN1P1*	profilin 1 pseudogene 1	4.04E-11	0.3673
7.	*AC005884.1*	Unmatched	4.36E-11	0.4013
8.	*CALM2P2*	calmodulin 2 pseudogene 2	4.36E-11	0.3855
9.	*HSPA8P1*	heat shock protein family A (Hsp70) member 8 pseudogene 1	4.36E-11	0.3791
10.	*RP11-490H24.5*	Unmatched	4.36E-11	0.3124
11.	*RP4-706A16.3*	Unmatched	4.36E-11	0.4548
12.	*TBC1D22A*	TBC1 domain family member 22A	4.36E-11	1.4314
13.	*EIF4A1P10*	eukaryotic translation initiation factor 4A1 pseudogene 10	4.66E-11	0.4609
14.	*HSP90AB3P*	heat shock protein 90 alpha family class B member 3, pseudogene	4.66E-11	0.3618
15.	*RP11-20O24.4*	Unmatched	6.43E-11	0.4450
16.	*RP11-1033A18.1*	Unmatched	7.00E-11	0.3813
17.	*EIF3FP3*	eukaryotic translation initiation factor 3 subunit F pseudogene 3	1.35E-10	0.4425
18.	*TRAPPC9*	trafficking protein particle complex 9	1.38E-10	1.5147
19.	*PDIA3P*	protein disulfide isomerase family A member 3 pseudogene 1	2.38E-10	0.4653
20.	*HSPA9P1*	heat shock protein family A (Hsp70) member 9 pseudogene 1	2.76E-10	0.4196
21.	*AC007238.1*	unmatched	3.62E-10	0.4215
22.	*HNRNPA1P7*	heterogeneous nuclear ribonucleoprotein A1 pseudogene 7	3.72E-10	0.4620
23.	*RP11-262D11.2*	Unmatched	4.68E-10	0.4851
24.	*RP11-159C21.4*	Unmatched	4.81E-10	0.3902
25.	*PABPC3*	poly(A) binding protein cytoplasmic 3	1.70E-09	0.4141
26.	*EEF1A1P6*	eukaryotic translation elongation factor 1 alpha 1 pseudogene 6	1.94E-09	0.4414
27.	*RP11-74E24.2*	Unmatched	1.94E-09	0.5369
28.	*GLUD2*	glutamate dehydrogenase 2	2.70E-09	0.4752
29.	*XRCC6P2*	X-ray repair cross complementing 6 pseudogene 2	2.89E-09	0.3728
30.	*HNRNPKP2*	heterogeneous nuclear ribonucleoprotein K pseudogene 2	3.13E-09	0.4239

31.	*EEF1A1P11*	eukaryotic translation elongation factor 1 alpha 1 pseudogene 11	8.40E-09	0.4479
32.	*UBA52P5*	ubiquitin A-52 residue ribosomal protein fusion product 1 pseudogene 5	8.40E-09	0.3967
33.	*RPL9P7*	ribosomal protein L9 pseudogene 7	9.10E-09	0.4136
34.	*PPP6R2*	protein phosphatase 6 regulatory subunit 2	9.52E-09	1.3609
35.	*HSP90B3P*	heat shock protein 90 beta family member 3, pseudogene	9.75E-09	0.3888
36.	*PSME1*	proteasome activator subunit 1	1.07E-08	0.5585
37.	*RP11-334L9.1*	Unmatched	1.37E-08	0.3330
38.	*RPS21P4*	ribosomal protein S21 pseudogene 4	1.37E-08	0.3759
39.	*AC138123.2*	Unmatched	1.38E-08	0.4073
40.	*CAP1P2*	CAP1 pseudogene 2	1.38E-08	0.4685
41.	*HNRNPKP4*	heterogeneous nuclear ribonucleoprotein K pseudogene 4	1.38E-08	0.4620
42.	*PTGES3P1*	prostaglandin E synthase 3 pseudogene 1	1.38E-08	0.5104
43.	*RPL9P8 (RPL9P9)*	ribosomal protein L9 pseudogene 8	1.38E-08	0.4179
44.	*HNRNPA1P10*	heterogeneous nuclear ribonucleoprotein A1 pseudogene 10	1.39E-08	0.4748
45.	*UBL5P2*	ubiquitin like 5 pseudogene 2	1.58E-08	0.2991
46.	*B3GNTL1*	UDP-GlcNAc:betaGal beta-1,3-N-acetylglucosaminyltransferase like 1	1.79E-08	1.6047
47.	hsa-mir-6723	Unmatched	2.78E-08	0.5456
48.	*TOM1L2*	target of myb1 like 2 membrane trafficking protein	3.63E-08	1.3864
49.	*MORF4L1P1*	mortality factor 4 like 1 pseudogene 1	3.98E-08	0.5354
50.	*RPL5P4*	ribosomal protein L5 pseudogene 4	4.39E-08	0.4647
51.	*EIF4BP3*	eukaryotic translation initiation factor 4B pseudogene 3	4.59E-08	0.4610
52.	*RPL39P3*	ribosomal protein L39 pseudogene 3	5.13E-08	0.3856
53.	*HSPA8P9*	heat shock protein family A (Hsp70) member 8 pseudogene 9	5.45E-08	0.3551
54.	*YWHAZP2*	tyrosine 3-monooxygenase/tryptophan 5-monooxygenase activation protein zeta pseudogene 2	5.45E-08	0.4631
55.	*EEF1A1P5*	eukaryotic translation elongation factor 1 alpha 1 pseudogene 5	6.53E-08	0.6110
56.	*CDC42P6*	cell division cycle 42 pseudogene 6	6.53E-08	0.4530
57.	*RPL3P4*	ribosomal protein L3 pseudogene 4	6.88E-08	0.4887
58.	*CTD-2373J19.1*	Unmatched	7.15E-08	0.4835
59.	*PTMAP5*	prothymosin alpha pseudogene 5	8.19E-08	0.5618
60.	*RP11-676M6.1*	Unmatched	8.19E-08	0.4978
61.	*CALM2P4*	calmodulin 2 pseudogene 4	8.91E-08	0.3755
62.	*RP11-627K11.1*	Unmatched	8.93E-08	0.4480
63.	*RPL7AP66*	ribosomal protein L7a pseudogene 66	9.71E-08	0.4847
64.	*MSNP1*	moesin pseudogene 1	9.82E-08	0.4805
65.	*RP11-680H20.1*	Unmatched	9.99E-08	0.4114
66.	*PSME2P2*	proteasome activator subunit 2 pseudogene 2	1.05E-07	0.3109
67.	*GAK*	cyclin G associated kinase	1.10E-07	1.2912

68.	*HNRNPA1P48*	heterogeneous nuclear ribonucleoprotein A1 pseudogene 48	1.20E-07	0.4560
69.	*RPL7AP30*	ribosomal protein L7a pseudogene 30	1.24E-07	0.3563
70.	*CTB-13H5.1*	Unmatched	1.41E-07	0.4177
71.	*HNRNPA1P35*	heterogeneous nuclear ribonucleoprotein A1 pseudogene 35	1.49E-07	0.3498
72.	*PTBP1P*	polypyrimidine tract binding protein 1 pseudogene	1.53E-07	0.4425
73.	*API5P1*	apoptosis inhibitor 5 pseudogene 1	1.57E-07	0.3469
74.	*SC22CB-1E7.1*	Unmatched	1.59E-07	0.4434
75.	*UBE2D3P1*	ubiquitin conjugating enzyme E2 D3 pseudogene 1	1.69E-07	0.4852
76.	*DYNC1I2P1*	dynein cytoplasmic 1 intermediate chain 2 pseudogene 1	1.83E-07	0.3828
77.	*AL162151.3*	Unmatched	1.94E-07	0.4310
78.	*RP11-286H14.4*	Unmatched	2.06E-07	0.4533
79.	*RPL9P8*	ribosomal protein L9 pseudogene 8	2.34E-07	0.4464
80.	*EEF1A1P13*	eukaryotic translation elongation factor 1 alpha 1 pseudogene 13	2.51E-07	0.5209
81.	*PABPC1P4*	poly(A) binding protein cytoplasmic 1 pseudogene 4	2.60E-07	0.4654
82.	*HNRNPUP1*	heterogeneous nuclear ribonucleoprotein U pseudogene 1	2.73E-07	0.4405
83.	*UPF1*	UPF1 RNA helicase and ATPase	2.82E-07	1.2468
84.	*RP11-416K24.2*	Unmatched	2.92E-07	0.2576
85.	*PTMAP2*	prothymosin alpha pseudogene 2	3.18E-07	0.4815
86.	*RPL7AP6*	ribosomal protein L7a pseudogene 6	3.20E-07	0.4192
87.	*ARPC3P1*	actin related protein 2/3 complex subunit 3 pseudogene 1	3.72E-07	0.3313
88.	*RPL3P2*	ribosomal protein L3 pseudogene 2	3.72E-07	0.4349
89.	*CTNNA1P1 (CTNNAP1)*	catenin alpha 1 pseudogene 1	3.84E-07	0.3255
90.	*HECTD4*	HECT domain E3 ubiquitin protein ligase 4	4.12E-07	1.3706
91.	*PTP4A2P1*	PTP4A2 pseudogene 1	4.59E-07	0.5002
92.	*WNK1*	WNK lysine deficient protein kinase 1	4.59E-07	1.2575
93.	*RPLP0P6*	ribosomal protein lateral stalk subunit P0 pseudogene 6	4.76E-07	0.5054
94.	*CTC-451P13.1*	Unmatched	4.77E-07	0.5126
95.	*MECP2*	methyl-CpG binding protein 2	4.77E-07	1.4360
96.	*CDS2*	CDP-diacylglycerol synthase 2	5.31E-07	1.2409
97.	*RP11-778D9.4*	Unmatched	5.31E-07	0.4437
98.	*EIF3LP1*	eukaryotic translation initiation factor 3 subunit L pseudogene 1	6.33E-07	0.4172
99.	*RP11-345J4.6*	Unmatched	6.99E-07	0.5361
100.	*KLRC1*	killer cell lectin like receptor C1	7.09E-07	0.3331
101.	*HSP90AB2P*	heat shock protein 90 alpha family class B member 2, pseudogene	7.83E-07	0.2673
102.	*BZW1P2*	basic leucine zipper and W2 domains 1 pseudogene 2	7.97E-07	0.4450
103.	*BRF1*	BRF1 RNA polymerase III transcription initiation factor subunit	8.40E-07	1.3745

104.	*BRD7P2*	bromodomain containing 7 pseudogene 2	8.46E-07	0.4541
105.	*TMSB10P1*	thymosin beta 10 pseudogene 1	8.76E-07	0.4217
106.	*ANXA2P2*	annexin A2 pseudogene 2	9.24E-07	0.4814
107.	*CFL1P4*	cofilin 1 pseudogene 4	9.88E-07	0.3852
108.	*AC005795.1*	Unmatched	1.01E-06	0.5572
109.	*RP11-216N14.7*	Unmatched	1.01E-06	2.0662
110.	*FTLP3*	ferritin light chain pseudogene 3	1.04E-06	0.5024
111.	*LPAR2*	lysophosphatidic acid receptor 2	1.04E-06	1.5448
112.	*RP11-556K13.1*	Unmatched	1.04E-06	0.4975
113.	*RP11-434O22.1*	Unmatched	1.16E-06	0.3615
114.	*AC016739.2*	Unmatched	1.27E-06	0.3293
115.	*DPP9*	dipeptidyl peptidase 9	1.56E-06	1.5269
116.	*PRRC2B*	proline rich coiled-coil 2B	1.56E-06	1.2727
117.	*NACA3P*	NACA family member 3, pseudogene	1.60E-06	0.4007
118.	*RPL4P5*	ribosomal protein L4 pseudogene 5	1.65E-06	0.4571
119.	*DGKD*	diacylglycerol kinase delta	1.78E-06	1.3415
120.	*RPL5P23*	ribosomal protein L5 pseudogene 23	1.78E-06	0.4059
121.	*RP11-12M9.3*	Unmatched	1.83E-06	0.4567
122.	*RP11-773H22.2*	Unmatched	1.83E-06	0.3240
123.	*POLR2A*	RNA polymerase II subunit A	1.93E-06	1.3440
124.	*RPL13AP25*	ribosomal protein L13a pseudogene 25	1.93E-06	0.5083
125.	*RP11-318C24.1*	Unmatched	1.94E-06	0.3135
126.	*RP3-417G15.1*	Unmatched	1.95E-06	0.4995
127.	*(OTUD4P1) HIN1L*	OTUD4 pseudogene 1	2.08E-06	0.4799
128.	*RP11-122C9.1*	Unmatched	2.10E-06	0.5222
129.	*RP1-102E24.1*	Unmatched	2.12E-06	0.3355
130.	*TCF25*	transcription factor 25	2.19E-06	1.3388
131.	*WASHC2A (FAM21A)*	WASH complex subunit 2A	2.21E-06	0.6062
132.	*CTD-2270N23.1*	Unmatched	2.22E-06	0.4548
133.	*RP11-265N6.3*	Unmatched	2.22E-06	0.3834
134.	*MAP4K2*	mitogen-activated protein kinase kinase kinase kinase 2	2.22E-06	1.5399
135.	*AC000089.3*	Unmatched	2.29E-06	0.3857
136.	*EIF3LP2*	eukaryotic translation initiation factor 3 subunit L pseudogene 2	2.33E-06	0.4574
137.	*RP11-274E7.2*	Unmatched	2.36E-06	0.3910
138.	*THRAP3P1*	thyroid hormone receptor associated protein 3 pseudogene 1	2.39E-06	0.4249
139.	*TYRO3P*	TYRO3P protein tyrosine kinase pseudogene	2.43E-06	2.1670
140.	*CTD-2554C21.1*	Unmatched	2.44E-06	0.4892
141.	*RPL18P13*	ribosomal protein L18 pseudogene 13	2.79E-06	0.3358
142.	*RP11-516A11.1*	Unmatched	2.89E-06	0.3476
143.	*SRP9P1*	signal recognition particle 9 pseudogene 1	2.89E-06	0.4477
144.	*SNORD62B*	small nucleolar RNA, C/D box 62B	2.93E-06	0.4316
145.	*HSPA8P7*	heat shock protein family A (Hsp70) member 8 pseudogene 7	2.98E-06	0.3053
146.	*NISCH*	Nischarin	3.18E-06	1.2319
147.	*RPL19P12*	ribosomal protein L19 pseudogene 12	3.18E-06	0.4243
148.	*NUP214*	nucleoporin 214	3.24E-06	1.2625

149.	*RAC1P2*	Rac family small GTPase 1 pseudogene 2	3.29E-06	0.4893
150.	*SNORD20*	small nucleolar RNA, C/D box 20	3.32E-06	0.3994
151.	*RPL13AP5*	ribosomal protein L13a pseudogene 5	3.76E-06	0.4760
152.	*AC022431.1*	Unmatched	3.79E-06	0.3990
153.	*RNU2-36P*	RNA, U2 small nuclear 36, pseudogene	3.85E-06	0.3880
154.	*EEF1DP1*	eukaryotic translation elongation factor 1 delta pseudogene 1	3.95E-06	0.4753
155.	*HK2P1*	hexokinase 2 pseudogene 1	4.10E-06	0.4017
156.	*RPS3AP5*	ribosomal protein S3A pseudogene 5	4.23E-06	0.4798
157.	*RP6-145B8.3*	Unmatched	4.32E-06	0.3607
158.	*HDAC5*	histone deacetylase 5	4.89E-06	1.4317
159.	*DNAH1*	dynein axonemal heavy chain 1	4.90E-06	1.4204
160.	*NATD1 (C17orf103)*	N-acetyltransferase domain containing 1	5.09E-06	1.6826
161.	*FCGR3B*	Fc fragment of IgG receptor IIIb	5.69E-06	0.3067
162.	*RPL13AP7*	ribosomal protein L13a pseudogene 7	5.88E-06	0.4587
163.	*INTS11 (CPSF3L)*	integrator complex subunit 11	5.95E-06	1.2458
164.	*RPL23AP2*	ribosomal protein L23a pseudogene 2	5.95E-06	0.4061
165.	*RP11-40C6.2*	Unmatched	6.01E-06	0.4510
166.	*PPIAP29*	peptidylprolyl isomerase A pseudogene 29	6.06E-06	0.4568
167.	*RP11-181C21.4*	Unmatched	6.06E-06	0.4121
168.	*SNORD62A*	small nucleolar RNA, C/D box 62A	6.12E-06	0.4386
169.	*RPL24P4*	ribosomal protein L24 pseudogene 4	6.24E-06	0.4241
170.	*RPL4P3*	ribosomal protein L4 pseudogene 3	7.69E-06	0.4131
171.	*CPT1A*	carnitine palmitoyltransferase 1A	7.72E-06	2.0161
172.	*DGKZP1*	diacylglycerol kinase zeta pseudogene 1	7.77E-06	0.4929
173.	*IQCN (KIAA1683)*	IQ motif containing N	8.08E-06	1.6856
174.	*RP11-592N21.1*	Unmatched	8.08E-06	0.4054
175.	*WASF4P*	WASP family member 4, pseudogene	8.50E-06	0.4786
176.	*AC004074.3*	Unmatched	8.76E-06	0.3216
177.	*HNRNPCP2*	heterogeneous nuclear ribonucleoprotein C pseudogene 2	9.35E-06	0.5607
178.	*RPL14P1*	ribosomal protein L14 pseudogene 1	9.35E-06	0.4505
179.	*MAT2B*	methionine adenosyltransferase 2B	9.37E-06	0.7523
180.	*RNU1-28P*	RNA, U1 small nuclear 28, pseudogene	9.53E-06	0.2861
181.	*AC010468.1*	Unmatched	9.54E-06	0.4572
182.	*RP13-104F24.3*	Unmatched	9.70E-06	0.4346
183.	*RP4-561L24.3*	Unmatched	9.82E-06	2.5831

Table S8. The set of 74 differentially expressed genes resulted from UVE-PLS analysis in seven patients with CVD compared to seven controls. Presented genes were ordered according to decreasing PLS coefficients. Gene names not assigned to gene symbols by HUGO Multi-symbol checker were termed as "Unmatched". Gene symbols in brackets are synonyms or previous gene symbols.

No.	Gene symbol	Gene name	PLS coefficient
1.	*TSC2*	TSC complex subunit 2	8.197E-04
2.	*TBC1D22A*	TBC1 domain family member 22A	7.572E-04
3.	*PPP6R2*	protein phosphatase 6 regulatory subunit 2	6.225E-04
4.	*HDAC5*	histone deacetylase 5	5.694E-04
5.	*UPF1*	UPF1 RNA helicase and ATPase	5.077E-04
6.	*CDS2*	CDP-diacylglycerol synthase 2	4.756E-04
7.	*PRRC2B*	proline rich coiled-coil 2B	4.693E-04
8.	*INTS11 (CPSF3L)*	integrator complex subunit 11	4.683E-04
9.	*ZNF236*	zinc finger protein 236	4.418E-04
10.	*HTT*	huntingtin	4.399E-04
11.	*SBF1*	SET binding factor 1	4.398E-04
12.	*WNK1*	WNK lysine deficient protein kinase 1	4.134E-04
13.	*ZZEF1*	zinc finger ZZ-type and EF-hand domain containing 1	3.782E-04
14.	*CHFR*	checkpoint with forkhead and ring finger domains	3.380E-04
15.	*SRSF2*	serine and arginine rich splicing factor 2	-3.257E-04
16.	*RP11-624D20.1*	Unmatched	-5.295E-04
17.	*RP11-141P6.1*	Unmatched	-5.310E-04
18.	*TOPORS*	TOP1 binding arginine/serine rich protein	-7.202E-04
19.	*RAC1P2*	Rac family small GTPase 1 pseudogene 2	-8.192E-04
20.	*UBE2D3P1*	ubiquitin conjugating enzyme E2 D3 pseudogene 1	-8.801E-04
21.	*PTP4A2P1*	PTP4A2 pseudogene 1	-9.014E-04
22.	*RNU4-6P*	RNA, U4 small nuclear 6, pseudogene	-9.029E-04
23.	*CTC-451P13.1*	Unmatched	-9.263E-04
24.	*AC083873.4*	Unmatched	-9.439E-04
25.	*BZW1P2*	basic leucine zipper and W2 domains 1 pseudogene 2	-9.598E-04
26.	*OTUD4P1 (HIN1L)*	OTUD4 pseudogene 1	-9.962E-04
27.	*EIF3LP2*	eukaryotic translation initiation factor 3 subunit L pseudogene 2	-9.995E-04
28.	*TPMTP1*	thiopurine S-methyltransferase pseudogene 1	-9.996E-04
29.	*PABPC1P4*	poly(A) binding protein cytoplasmic 1 pseudogene 4	-1.031E-03
30.	*MORF4L1P1*	mortality factor 4 like 1 pseudogene 1	-1.045E-03
31.	*RP11-318C24.1*	Unmatched	-1.062E-03
32.	*RP11-74E24.2*	Unmatched	-1.067E-03
33.	*RPL7AP66*	ribosomal protein L7a pseudogene 66	-1.089E-03
34.	*PTBP1P*	polypyrimidine tract binding protein 1 pseudogene	-1.095E-03
35.	*HNRNPUP1*	heterogeneous nuclear ribonucleoprotein U pseudogene 1	-1.106E-03
36.	*HNRNPKP4*	heterogeneous nuclear ribonucleoprotein K pseudogene 4	-1.120E-03
37.	*RP11-680H20.1*	Unmatched	-1.155E-03
38.	*HNRNPKP2*	heterogeneous nuclear ribonucleoprotein K pseudogene 2	-1.163E-03

39.	*CTB-13H5.1*	Unmatched	-1.175E-03
40.	*API5P1*	apoptosis inhibitor 5 pseudogene 1	-1.204E-03
41.	*RP11-334L9.1*	Unmatched	-1.206E-03
42.	*RP11-676M6.1*	Unmatched	-1.208E-03
43.	*EEF1A1P13*	eukaryotic translation elongation factor 1 alpha 1 pseudogene 13	-1.211E-03
44.	*HNRNPA1P35*	heterogeneous nuclear ribonucleoprotein A1 pseudogene 35	-1.223E-03
45.	*HNRNPA1P10*	heterogeneous nuclear ribonucleoprotein A1 pseudogene 10	-1.227E-03
46.	*HNRNPA1P7*	heterogeneous nuclear ribonucleoprotein A1 pseudogene 7	-1.232E-03
47.	*PDIA3P1 (PDIA3P)*	protein disulfide isomerase family A member 3 pseudogene 1	-1.240E-03
48.	*RPL9P8*	ribosomal protein L9 pseudogene 8	-1.253E-03
49.	*AL162151.3*	Unmatched	-1.258E-03
50.	*ARPC3P1*	actin related protein 2/3 complex subunit 3 pseudogene 1	-1.272E-03
51.	*EIF4A1P10*	eukaryotic translation initiation factor 4A1 pseudogene 10	-1.286E-03
52.	*RPL9P8 (RPL9P9)*	ribosomal protein L9 pseudogene 8	-1.302E-03
53.	*EEF1A1P19*	eukaryotic translation elongation factor 1 alpha 1 pseudogene 19	-1.305E-03
54.	*UBA52P5*	ubiquitin A-52 residue ribosomal protein fusion product 1 pseudogene 5	-1.306E-03
55.	*EEF1A1P11*	eukaryotic translation elongation factor 1 alpha 1 pseudogene 11	-1.369E-03
56.	*EEF1A1P6*	eukaryotic translation elongation factor 1 alpha 1 pseudogene 6	-1.375E-03
57.	*RP4-706A16.3*	Unmatched	-1.394E-03
58.	*AC007238.1*	Unmatched	-1.398E-03
59.	*HSPA9P1*	heat shock protein family A (Hsp70) member 9 pseudogene 1	-1.414E-03
60.	*RPL9P7*	ribosomal protein L9 pseudogene 7	-1.417E-03
61.	*AC138123.2*	Unmatched	-1.422E-03
62.	*EIF3FP3*	eukaryotic translation initiation factor 3 subunit F pseudogene 3	-1.423E-03
63.	*PFN1P1*	profilin 1 pseudogene 1	-1.457E-03
64.	*PABPC3*	poly(A) binding protein cytoplasmic 3	-1.468E-03
65.	*AC005884.1*	Unmatched	-1.488E-03
66.	*RP11-1033A18.1*	Unmatched	-1.495E-03
67.	*CALM2P2*	calmodulin 2 pseudogene 2	-1.498E-03
68.	*RP11-490H24.5*	Unmatched	-1.508E-03
69.	*RPS21P4*	ribosomal protein S21 pseudogene 4	-1.531E-03
70.	*XRCC6P2*	X-ray repair cross complementing 6 pseudogene 2	-1.535E-03
71.	*RP11-159C21.4*	Unmatched	-1.552E-03
72.	*HSPA8P1*	heat shock protein family A (Hsp70) member 8 pseudogene 1	-1.575E-03
73.	*AC078899.1*	Unmatched	-1.586E-03
74.	*RP11-16F15.1*	Unmatched	-2.068E-03

Table S9. Results of ROC analysis for 62 genes selected as indicative for CVD.

Gene	ROC-AUC[1]	Threshold	Specificity	Sensitivity	Accuracy	Positive Predictive Value	Negative Predictive Value
AC005884.1	1.000	6.294	1.000	1.000	1.000	1.000	1.000
AC007238.1	1.000	5.737	1.000	1.000	1.000	1.000	1.000
AC078899.1	1.000	6.988	1.000	1.000	1.000	1.000	1.000
AC138123.2	1.000	7.109	1.000	1.000	1.000	1.000	1.000
AL162151.3	1.000	6.514	1.000	1.000	1.000	1.000	1.000
API5P1	1.000	4.669	1.000	1.000	1.000	1.000	1.000
ARPC3P1	1.000	4.852	1.000	1.000	1.000	1.000	1.000
BZW1P2	1.000	6.206	1.000	1.000	1.000	1.000	1.000
CALM2P2	1.000	5.342	1.000	1.000	1.000	1.000	1.000
CDS2	1.000	10.036	1.000	1.000	1.000	1.000	1.000
CTB-13H5.1	1.000	7.446	1.000	1.000	1.000	1.000	1.000
CTC-451P13.1	1.000	5.822	1.000	1.000	1.000	1.000	1.000
EEF1A1P11	1.000	9.226	1.000	1.000	1.000	1.000	1.000
EEF1A1P13	1.000	9.601	1.000	1.000	1.000	1.000	1.000
EEF1A1P19	1.000	8.575	1.000	1.000	1.000	1.000	1.000
EEF1A1P6	1.000	11.713	1.000	1.000	1.000	1.000	1.000
EIF3FP3	1.000	7.217	1.000	1.000	1.000	1.000	1.000
EIF3LP2	1.000	5.309	1.000	1.000	1.000	1.000	1.000
EIF4A1P10	1.000	7.476	1.000	1.000	1.000	1.000	1.000
HDAC5	1.000	9.888	1.000	1.000	1.000	1.000	1.000
HNRNPA1P10	1.000	7.474	1.000	1.000	1.000	1.000	1.000
HNRNPA1P35	1.000	5.228	1.000	1.000	1.000	1.000	1.000
HNRNPA1P7	1.000	7.641	1.000	1.000	1.000	1.000	1.000
HNRNPKP2	1.000	6.274	1.000	1.000	1.000	1.000	1.000
HNRNPKP4	1.000	7.761	1.000	1.000	1.000	1.000	1.000
HNRNPUP1	1.000	5.304	1.000	1.000	1.000	1.000	1.000
HSPA8P1	1.000	6.352	1.000	1.000	1.000	1.000	1.000
HSPA9P1	1.000	5.744	1.000	1.000	1.000	1.000	1.000
INTS11 (CPSF3L)	1.000	9.544	1.000	1.000	1.000	1.000	1.000
MORF4L1P1	1.000	7.438	1.000	1.000	1.000	1.000	1.000
OTUD4P1 (HIN1L)	1.000	5.204	1.000	1.000	1.000	1.000	1.000
PABPC1P4	1.000	7.550	1.000	1.000	1.000	1.000	1.000
PABPC3	1.000	8.575	1.000	1.000	1.000	1.000	1.000
PDIA3P1 (PDIA3P)	1.000	7.152	1.000	1.000	1.000	1.000	1.000
PFN1P1	1.000	6.578	1.000	1.000	1.000	1.000	1.000
PPP6R2	1.000	9.867	1.000	1.000	1.000	1.000	1.000
PRRC2B	1.000	11.692	1.000	1.000	1.000	1.000	1.000
PTBP1P	1.000	5.423	1.000	1.000	1.000	1.000	1.000
PTP4A2P1	1.000	5.670	1.000	1.000	1.000	1.000	1.000
RAC1P2	0.980	6.007	0.857	1.000	0.929	0.875	1.000
RP11-1033A18.1	1.000	7.100	1.000	1.000	1.000	1.000	1.000
RP11-159C21.4	1.000	8.052	1.000	1.000	1.000	1.000	1.000
RP11-16F15.1	1.000	7.326	1.000	1.000	1.000	1.000	1.000
RP11-318C24.1	0.980	4.523	0.857	1.000	0.929	0.875	1.000
RP11-334L9.1	1.000	4.450	1.000	1.000	1.000	1.000	1.000
RP11-490H24.5	1.000	4.660	1.000	1.000	1.000	1.000	1.000
RP11-676M6.1	1.000	5.758	1.000	1.000	1.000	1.000	1.000
RP11-680H20.1	1.000	6.427	1.000	1.000	1.000	1.000	1.000

RP11-74E24.2	1.000	6.302	1.000	1.000	1.000	1.000	1.000
RP4-706A16.3	1.000	8.025	1.000	1.000	1.000	1.000	1.000
RPL7AP66	1.000	6.241	1.000	1.000	1.000	1.000	1.000
RPL9P7	1.000	7.833	1.000	1.000	1.000	1.000	1.000
RPL9P8	1.000	7.955	1.000	1.000	1.000	1.000	1.000
RPL9P9	1.000	7.906	1.000	1.000	1.000	1.000	1.000
RPS21P4	1.000	6.861	1.000	1.000	1.000	1.000	1.000
TBC1D22A	1.000	9.526	1.000	1.000	1.000	1.000	1.000
TSC2	1.000	9.855	1.000	1.000	1.000	1.000	1.000
UBA52P5	1.000	5.889	1.000	1.000	1.000	1.000	1.000
UBE2D3P1	1.000	6.522	1.000	1.000	1.000	1.000	1.000
UPF1	1.000	10.151	1.000	1.000	1.000	1.000	1.000
WNK1	1.000	11.834	1.000	1.000	1.000	1.000	1.000
XRCC6P2	1.000	6.147	1.000	1.000	1.000	1.000	1.000

[1]Area under ROC curve.

Table S10: Correlation analysis between age and expression of 62 selected genes in CVD group. Genes were ordered according to increasing *P* value.

Gene	Correlation coefficient	*P* value
HSPA8P1	-0.82	0.024
PTBP1P	-0.80	0.031
TSC2	-0.79	0.033
UBA52P5	-0.77	0.043
HIN1L	-0.73	0.061
PRRC2B	-0.70	0.078
MORF4L1P1	-0.64	0.124
TBC1D22A	-0.64	0.124
BZW1P2	-0.63	0.128
AC007238.1	-0.63	0.130
RP11-676M6.1	-0.61	0.145
RAC1P2	0.61	0.149
CALM2P2	-0.60	0.155
RP11-490H24.5	0.59	0.164
RPS21P4	-0.58	0.170
HNRNPA1P10	-0.58	0.170
RP11-159C21.4	-0.55	0.205
CTC-451P13.1	0.53	0.222
AC005884.1	0.49	0.264
EEF1A1P19	0.47	0.290
HNRNPKP2	-0.45	0.312
HDAC5	0.43	0.335
RP11-334L9.1	-0.40	0.368
RP11-318C24.1	-0.40	0.374
RP11-16F15.1	-0.38	0.402
PABPC1P4	-0.36	0.426
EEF1A1P6	-0.34	0.455
HSPA9P1	0.33	0.474
RP11-1033A18.1	-0.32	0.486
RPL7AP66	-0.30	0.516
HNRNPUP1	-0.30	0.520
PFN1P1	0.29	0.531
HNRNPA1P35	0.29	0.533
PABPC3	-0.28	0.542
EIF4A1P10	0.28	0.549
RPL9P8	-0.28	0.549
AC078899.1	-0.26	0.570
RP4-706A16.3	0.26	0.570
PPP6R2	-0.25	0.583

PDIA3P	0.23	0.626
CPSF3L	0.22	0.630
AL162151.3	-0.22	0.632
XRCC6P2	-0.22	0.633
UBE2D3P1	0.22	0.642
UPF1	-0.21	0.647
API5P1	-0.20	0.664
HNRNPKP4	0.20	0.675
ARPC3P1	0.19	0.678
RP11-74E24.2	-0.17	0.714
RPL9P7	-0.16	0.724
CDS2	0.16	0.730
EIF3LP2	0.14	0.763
WNK1	0.14	0.771
RPL9P9	-0.13	0.780
CTB-13H5.1	0.10	0.824
AC138123.2	0.09	0.847
EEF1A1P11	0.07	0.879
RP11-680H20.1	-0.04	0.925
EEF1A1P13	-0.01	0.976
EIF3FP3	-0.01	0.989
PTP4A2P1	0.00	0.995
HNRNPA1P7	0.00	0.998

Table S11: Twelve experimentally validated miRNA:gene pairs found among 31 miRNAs and 62 genes indicative for CVD using multiMiR package. PAR-CLIP - Photoactivatable Ribonucleoside-Enhanced Crosslinking and Immunoprecipitation, HITS-CLIP - High-Throughput Sequencing of RNA isolated by Cross-Linking Immunoprecipitation, CLASH - Cross-linking, Ligation, and Sequencing of Hybrids.

	**Database**	**miRNA ID**	**Gene symbol**	**Method**
1.	mirtarbase	hsa-miR-106b-3p	*WNK1*	CLASH
2.		hsa-miR-183-5p	*WNK1*	CLASH
3.		hsa-let-7b-3p	*WNK1*	PAR-CLIP
4.		hsa-miR-590-3p	*WNK1*	HITS-CLIP
5.		hsa-miR-19a-3p	*WNK1*	HITS-CLIP
6.		hsa-miR-181a-2-3p	*WNK1*	HITS-CLIP
7.		hsa-miR-92a-3p	*PPP6R2*	CLASH
8.		hsa-miR-34a-5p	*PRRC2B*	CLASH
9.		hsa-miR-92a-3p	*PRRC2B*	PAR-CLIP, HITS-CLIP, CLASH
10.		hsa-miR-25-3p	*PRRC2B*	PAR-CLIP
11.		hsa-miR-497-3p	*PRRC2B*	PAR-CLIP
12.		hsa-miR-33a-5p	*PABPC3*	PAR-CLIP, HITS-CLIP

Table S12: Top 10%predicted miRNA:gene pairs found among 31 miRNAs and 62 genes indicative for CVD using multiMiR package.

	Database	miRNA ID	Gene symbol	Database-specific probability value
1.	diana_microt	hsa-miR-454-3p	*WNK1*	0.999
2.		hsa-miR-548d-3p	*CDS2*	0.961
3.		hsa-miR-548aa	*CDS2*	0.916
4.		hsa-miR-548t-3p	*CDS2*	0.916
5.		hsa-miR-1277-5p	*CDS2*	0.916
6.		hsa-miR-548ac	*CDS2*	0.908
7.		hsa-miR-548ac	*PRRC2B*	0.888
8.		hsa-miR-19a-3p	*WNK1*	0.865
9.		hsa-miR-548d-3p	*PRRC2B*	0.864
10.		hsa-miR-590-3p	*PABPC3*	0.854
11.		hsa-miR-590-3p	*WNK1*	0.832
12.		hsa-miR-92a-3p	*PRRC2B*	0.828
13.		hsa-miR-576-3p	*WNK1*	0.819
14.		hsa-miR-548ac	*WNK1*	0.815
15.		hsa-miR-548t-3p	*PRRC2B*	0.811
16.		hsa-miR-548aa	*PRRC2B*	0.811
17.		hsa-miR-454-3p	*PABPC3*	0.806
18.	elmmo	hsa-let-7b-3p	*WNK1*	0.846
19.		hsa-miR-19a-3p	*WNK1*	0.82
20.		hsa-miR-96-5p	*TBC1D22A*	0.806
21.		hsa-miR-92a-3p	*PRRC2B*	0.802
22.		hsa-miR-96-5p	*WNK1*	0.738
23.		hsa-miR-19a-3p	*PPP6R2*	0.735
24.		hsa-miR-454-3p	*WNK1*	0.717
25.		hsa-miR-25-3p	*PRRC2B*	0.676
26.		hsa-miR-497-3p	*PRRC2B*	0.561
27.		hsa-miR-454-3p	*PPP6R2*	0.54
28.		hsa-let-7b-3p	*PRRC2B*	0.524
29.		hsa-miR-590-3p	*PABPC3*	0.492
30.		hsa-miR-590-3p	*WNK1*	0.491
31.	microcosm	hsa-miR-590-3p	*PABPC3*	18.5626
32.		hsa-miR-590-3p	*PABPC3*	18.5355
33.	miranda	hsa-miR-576-3p	*CDS2*	-1.0983
34.		hsa-miR-206	*WNK1*	-1.1794
35.		hsa-let-7b-3p	*WNK1*	-1.2827
36.	mirdb	hsa-miR-454-3p	*WNK1*	94.25965
37.		hsa-miR-454-3p	*WNK1*	94.2407
38.	pita	hsa-miR-34a-5p	*CDS2*	-10.96
39.		hsa-miR-34a-5p	*TBC1D22A*	-11.21
40.		hsa-miR-92a-3p	*PRRC2B*	-11.38
41.		hsa-miR-92a-3p	*TBC1D22A*	-11.38
42.		hsa-miR-769-5p	*HDAC5*	-11.51
43.		hsa-miR-34a-5p	*PPP6R2*	-12.7

44.		hsa-miR-25-3p	*TBC1D22A*	-12.78
45.		hsa-miR-34a-5p	*HDAC5*	-14.78
46.		hsa-miR-206	*PRRC2B*	-14.83
47.	targetscan	hsa-miR-19a-3p	*PPP6R2*	-0.243
48.		hsa-miR-25-3p	*PRRC2B*	-0.266
49.		hsa-miR-454-3p	*PPP6R2*	-0.31
50.		hsa-miR-454-3p	*PABPC3*	-0.317
51.		hsa-miR-128-3p	*WNK1*	-0.362
